# Efficient and scalable training set generation for automated pollen monitoring with Hirst-type samplers

**DOI:** 10.1038/s41598-025-31646-2

**Published:** 2025-12-17

**Authors:** András Biricz, Donát Magyar, Björn Gedda, Antonio Spanu, János Fillinger, Adrián Pesti, István Csabai, Péter Pollner

**Affiliations:** 1 Department of Physics of Complex Systems, ELTE Eötvös Loránd University, Budapest, Hungary; 2 National Center for Public Health and Pharmacy, Budapest, Hungary; 3 The Palynological Laboratory at the Swedish Museum of Natural History, Stockholm, Sweden; 4 INRAE, UR 546 BioSP, Site Agroparc, Avignon, France; 5 National Korányi Institute for Pulmonology, Budapest, Hungary; 6 Department of Pathology, Forensic and Insurance Medicine, Semmelweis University, Budapest, Hungary; 7 Data-Driven Health Division, National Laboratory for Health Security, Health Services Management Training Centre, Budapest, Hungary; 8 Department of Biological Physics, ELTE Eötvös Loránd University, Budapest, Hungary

**Keywords:** Airborne allergen analysis, Automated pollen detection, Deep learning, Hirst-type sampler, Open-vocabulary object detection, Pollen monitoring, Vision Transformer, Ecology, Computational biology and bioinformatics, Microscopy

## Abstract

Automated pollen detection is essential for ecological monitoring, allergy forecasting, and biodiversity research. However, existing methods rely heavily on manual or semi-automated annotations, limiting scalability and broader applicability. We introduce a highly automated training dataset generation pipeline that combines one-shot detection with systematic refinement, producing tens of thousands of high-quality annotations from bright-field microscopy while significantly reducing manual effort and annotation costs. Using multi-regional datasets from France, Hungary, and Sweden, we trained object detection models on seven pollen taxa and evaluated their performance on both external pure and mixed species slides and real-world airborne samples. We assessed the reusability of pretrained vision models for pollen detection, aiming to reduce the need for extensive retraining. Using linear probing, we identified foundational Vision Transformers (ViTs) as the most effective feature extractors and integrated them into Faster R-CNN detection models. We benchmarked these models against ResNet50, a widely adopted backbone in biological imaging. On held-out regions of the training datasets, our models achieved high performance in both classification and detection tasks. On independent reference slides from other datasets, ViTs continued to outperform ResNet50 in classification. However, in full object detection and under real deployment conditions, ResNet50-based models remained competitive and achieved the highest accuracy for detecting *Ambrosia*, a major allergen with public health significance. Cross-dataset generalization remains a challenge, underscoring the need for domain adaptation techniques such as stain normalization and data augmentation. This study establishes a scalable framework for AI-assisted pollen monitoring, supporting large-scale slide digitization and enabling applications in long-term ecological research, allergen surveillance, and automated biodiversity assessment.

## Introduction

Automated pollen analysis supports accurate identification of airborne pollen grains, with far-reaching implications for public health^1,2^, paleoclimatology^3^, and pollination ecology^4^. The global rise in pollen allergies, projected to increase due to climate change^5^, underscores the need for real-time, scalable monitoring systems. Despite established guidelines and standards^6,7^, conventional identification methods remain labor-intensive, time-consuming, and implicitly subjective, hindering the efficiency of pollen forecasting and allergy management^8^. To address these challenges, AI-powered automation offers a transformative opportunity to deliver scalable, high-throughput, consistently performing pollen monitoring, crucial for mitigating health risks, improving forecasting accuracy, and strengthening environmental resilience^9,10^ - particularly for real-time allergy warning systems and long-term ecological studies.

However, automating pollen monitoring remains challenging due to hardware constraints, high costs, and proprietary limitations^8^. Although some commercial systems exist, many remain inaccessible or require further validation^11,12^. Other emerging technologies, such as laser scattering^13^, lidar-based remote sensing^14^, chemical detection^15^, multispectral imaging flow cytometry^16^, and DNA analysis^17^, show promise but remain cost-prohibitive and restricted to specialized proprietary systems^18^.

The Hirst-type pollen trap remains the global standard for airborne pollen monitoring, valued for its cost-effectiveness, robustness, and widespread adoption in national aerobiological networks^19^. However, its manual, expert-led processing is labor-intensive, subjective, and limits scalability, creating a significant bottleneck for both real-time monitoring and retrospective analysis^6^. For instance, Hungary alone holds an archive of over 140,000 air-sample slides spanning 30 years; France has maintained nationwide Hirst-type observations since the late 1980s^20^, and Sweden’s national programme coordinated by the Swedish Museum of Natural History, provides long-running observations^21^ - together representing a vast but underutilized resource that automation could unlock for long-term ecological studies. On the other hand, operational deployments would also require slide preparation and high-resolution digitization - each incurring non-trivial time, compute, and storage at pollen monitoring network scale.

Recent advances in image recognition and object detection, including YOLO^22^ and Faster R-CNN-based models^23–25^, have transformed bright-field microscopy applications, significantly facilitating automation^26–29^. These advances reflect a broader shift in AI, powered by deep learning architectures such as Residual Networks (ResNets)^30^, Vision Transformers (ViTs)^31^, and hybrid transformer-based models^32–34^. These models have achieved strong performance across many image-analysis tasks^35^, establishing new benchmarks in automated pollen detection^36–38^.

Despite recent advances, the preparation of training datasets remains a critical bottleneck in automated pollen analysis, requiring substantial human effort. Existing methods range from fully manual expert annotation^39^ to semi-automated approaches^40^ and traditional image processing techniques^29^. However, these approaches often lack scalability, limiting their practical application in high-throughput monitoring.

A promising solution lies in the integration of open-vocabulary object detectors, which can improve scalability and reduce annotation costs^41,42^. In particular, the Vision Transformer for Open-World Localization (OWL-ViT)^43^ enables one-shot detection, identifying previously unseen pollen species with minimal supervision. By leveraging this capability, AI-driven pipelines can generate robust, scalable datasets, significantly reducing reliance on manual annotation and traditional image thresholding methods while addressing persistent challenges in generalization and training data costs. In practical terms, the open-vocabulary detector is a pretrained vision-language foundational model^43^ that, when prompted by a text label or a single exemplar image, localizes candidate pollen grains. It can be coupled with conventional pre- and post-processing and serves as the initiator for training set generation.

In this study we introduce a two-stage, AI-based workflow for pollen microscopy as a research-stage framework. First, the system automatically harvests training examples from pure-species slides with minimal supervision; second, the resulting dataset is used to train pollen-detection models, focusing on downstream efficiency. In the second stage, we reuse pretrained, general-purpose vision encoders (LVD-ViT) as feature backbones, so broad visual representations can be leveraged when labeled data and compute are limited. These encoders plug into standard detectors (e.g., Faster R-CNN^23^), enabling efficient training and improving transfer across staining protocols, microscopes, and regions.

We evaluate the auto-generated data by training several detectors and testing them on independent slides from France, Hungary, and Sweden that differ in staining protocols, microscopes, and climate-conditions encountered in practice that probe the limits of cross-region transfer. Therefore, we prioritize cross-region rather than solely intra-dataset evaluation. By cutting the annotation burden from hours or days to minutes, the pipeline streamlines routine analysis, providing a practical entry point for AI-assisted airborne pollen monitoring and laying the groundwork for broader integration of AI tools in aerobiological research.

## Results

**Fig. 1:**
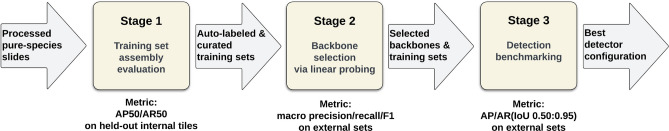
Study design and evaluation flow. The pipeline is summarized in three stages: (1) *training set assembly* from pure-species slides; (2) *backbone selection* by linear probing; and (3) *detection benchmarking* on external slides. Metrics are AP50/AR50 (Stage 1), macro precision/recall/F1 (Stage 2), and AP/AR at IoU 0.50-0.95 (Stage 3).

We evaluated our pipeline in three stages (see Fig. 1), with a particular focus on generalization across external datasets to ensure real-world applicability beyond the training domain. Throughout, external denotes evaluation on slides from different slides and/or regions - including Hungary, Sweden, and, where label overlap permits, France (see Supplementary Fig. S1). First, we assessed the quality of automated training set generation using the Vision Transformer for Open-World Localization (OWL-ViT)^43^, including the effects of annotation refinement and patch size variations (Section Automated training set assembly). Second, we analyzed the feature extraction capabilities of Vision Transformer (ViT) backbones through linear probing to evaluate their adaptability to pollen detection (Section Evaluation of foundational backbones). Third, we benchmarked object detection performance by comparing ViT-based Faster R-CNN models to conventional architectures across multi-regional datasets (Section Performance of object detection models), and tested robustness on real-world airborne pollen samples (Section Real-world validation on air samples).

### Automated training set assembly

Our automated annotation pipeline enabled large-scale, high-throughput labeling of pure-species pollen slides with limited expert oversight (exemplar selection, threshold tuning, brief visual checks), while maintaining high annotation quality (Fig. 4). Using OWL-ViT for one-shot detection, followed by statistical filtering and refinement with YOLOS-Tiny^44^, the pipeline efficiently generated curated training datasets.

To evaluate annotation accuracy and the contribution of each refinement stage, we applied COCO-style detection metrics to predictions on internal test regions (Table 1). Across all datasets and patch sizes, Average Precision (AP) and Average Recall (AR) at an Intersection over Union (IoU) threshold of 0.50 improved consistently following refinement.

**Table 1:** Evaluation of the automated training set assembly across Hungarian, Swedish, and Mediterranean datasets, with performance metrics computed on non-overlapping internal test tiles. Annotation counts are shown at three stages-initial (Init., OWL-ViT predictions), filtered (RGB-based outlier removal), and refined (YOLOS-based reannotation)-for two patch sizes (518 px and 896 px). Detection performance is reported using Average Precision (AP50) and Average Recall (AR50) at IoU = 0.50, both before and after refinement. Bolded values indicate the final refined datasets used for object detector training. To ensure fair evaluation, only pollen grains with at least 80% of their bounding box contained within a tile were included, avoiding penalization for partially visible instances at tile boundaries.

Patch Size	Dataset	Initial	Filtered	Refined	AP50 (Init.)	AP50 (Ref.)	AR50 (Init.)	AR50 (Ref.)
518	Hungarian	29,871	21,845	**28,586**	0.552	**0.829**	0.938	**0.938**
	Swedish	39,897	30,009	**38,378**	0.551	**0.570**	0.766	**0.748**
	Mediterranean	8,768	5,944	**9,232**	0.540	**0.658**	0.636	**0.707**
896	Hungarian	30,598	21,192	**32,218**	0.689	**0.755**	0.811	**0.811**
	Swedish	48,814	33,978	**48,529**	0.585	**0.712**	0.676	**0.791**
	Mediterranean	9,175	6,129	**9,325**	0.572	**0.549**	0.614	**0.564**

Following YOLOS-based refinement, nearly all datasets exhibited consistent improvements in detection accuracy. For the Hungarian dataset-characterized by high-quality input slides with minimal object overlap-AP50 increased from 0.552 to 0.829 for 518-pixel patches and from 0.689 to 0.755 for 896-pixel patches, reflecting effective noise removal and instance recovery. Swedish and Mediterranean datasets showed more modest gains, particularly in recall, likely due to increased variability in sample quality and denser pollen grain distributions (see Sources and collection). To ensure a fair and consistent evaluation, internal testing was performed on non-overlapping tiles, and only pollen grains with at least 80% of their expert-annotated bounding box area within a single tile were considered. This constraint reduced boundary artifacts and highlighted model limitations in crowded or ambiguous regions.

Annotation count trends further supported the pipeline’s effectiveness. Initial detections were filtered to remove artifacts, debris, and background noise. YOLOS refinement then recovered valid instances and improved localization, yielding final annotation sets that closely matched-or exceeded-the original counts. This iterative improvement is critical for training robust models, as it enhances data quality without requiring extensive manual annotation.

Given the high-resolution imaging ($40\times$ magnification), individual pollen grains typically span  200 pixels in diameter, making patch size a key factor in balancing detection accuracy with computational efficiency. Patch sizes were selected primarily for compatibility with backbone architectures, aligning with the native input dimensions of Vision Transformers (ViTs) and Faster R-CNN models. Among the tested sizes, 518 and 896 pixels offered the best trade-offs between model readiness and runtime. An ablation study (see Supplementary Table S3) evaluated annotation consistency across patch sizes from 518 to 1554 pixels. Smaller patches improved localization but were more sensitive to edge effects, while larger patches provided more context at the cost of computational overhead. Overall, 896-pixel patches yielded the most stable and accurate results, with 518-pixel patches remaining a viable alternative for speed-critical scenarios. These two configurations were used throughout the rest of the study to benchmark classifier and detector performance under consistent, architecture-aware conditions.

### Evaluation of foundational backbones

The performance of object detection models is closely tied to the quality of their feature extractors, or backbones. To assess how well different backbone architectures generalize across regions, we conducted a linear probing analysis, evaluating classification accuracy on external test datasets. This method isolates the feature extraction component-excluding localization-specific factors-providing a clean benchmark for backbone effectiveness prior to integration into full detection pipelines (Fig. 5).

We focused on external evaluation because internal test results-drawn from held-out regions of the same slides-often yield near-perfect scores, offering limited insight into real-world generalization. By contrast, external datasets introduce meaningful variation in staining, imaging, and acquisition conditions, offering a more realistic assessment of backbone robustness.

**Table 2:** Linear probing evaluation of foundational vision backbones on external test datasets. Models trained on Hungarian slides were evaluated on external slides from Hungary, Sweden, and France, while models trained on Swedish slides were tested on Swedish and French data. External results aggregate slides from Hungary, Sweden, and-where label overlap permitted-France; French slides were used for testing only. All test slides shared the same taxonomic labels as their corresponding training sets, allowing performance differences to reflect true generalization under domain shifts, rather than class mismatches. Results are reported as mean ± standard error computed over five cross-validation folds, based on macro-averaged precision, recall, and F1-score for each fold. LVD-ViT-L consistently outperformed other backbones, demonstrating superior feature extraction and generalization. RN50 models underperformed, highlighting the limitations of convolutional architectures for cross-domain pollen classification.

Training dataset	Model	Precision	Recall	F1-Score
Hungarian-trained models	LVD-ViT-S	0.924 ± 0.001	0.761 ± 0.009	0.804 ± 0.007
	LVD-ViT-B	0.920 ± 0.002	0.718 ± 0.025	0.77 ± 0.02
	**LVD-ViT-L**	**0.937 ± 0.001**	**0.872 ± 0.005**	**0.890 ± 0.004**
	UNI-ViT-L	0.921 ± 0.001	0.73 ± 0.01	0.78 ± 0.01
	RN50 (DINO)	0.4 ± 0.2	0.115 ± 0.002	0.028 ± 0.003
	RN50 (COCO)	0.78 ± 0.01	0.5 ± 0.1	0.55 ± 0.10
Swedish-trained models	LVD-ViT-S	**0.58 ± 0.02**	0.468 ± 0.009	0.46 ± 0.01
	LVD-ViT-B	0.47 ± 0.01	0.38 ± 0.01	0.39 ± 0.01
	**LVD-ViT-L**	0.543 ± 0.004	**0.52 ± 0.01**	**0.510 ± 0.008**
	UNI-ViT-L	0.505 ± 0.002	0.45 ± 0.01	0.462 ± 0.009
	RN50 (DINO)	0.449 ± 0.015	0.217 ± 0.006	0.10 ± 0.01
	RN50 (COCO)	0.35 ± 0.08	0.18 ± 0.06	0.15 ± 0.06

Table 2 presents the classification performance of ViT backbones-ViT-Small (ViT-S), ViT-Base (ViT-B), and ViT-Large (ViT-L)-pretrained on large-scale datasets (LVD, UNI), alongside ResNet50 (RN50) variants, including COCO-pretrained and DINO self-supervised^45^. External evaluation used slides from Hungary, Sweden, and - where label overlap permitted - France (see Supplementary Fig. S1).

LVD-ViT-L consistently demonstrated superior classification performance, particularly when trained on the Hungarian dataset, achieving 0.937 ± 0.001 macro-averaged precision, 0.872 ± 0.005 macro-averaged recall (equivalent to 87.2 ± 0.5% balanced accuracy), and 0.890 ± 0.004 F1-score. These results underscored its ability to extract fine-grained pollen features, which is particularly critical for distinguishing morphologically similar species such as *Ambrosia* and *Iva*.

For models trained on the Swedish dataset, LVD-ViT-L continued to outperform smaller ViT variants and RN50 backbones, attaining a recall of 0.52 ± 0.01 (balanced accuracy of 52 ± 1.0%) and an F1-score of 0.510 ± 0.008. However, the lower overall performance for Swedish-trained models reflects the increased complexity of detecting multiple species in highly diverse, mixed-quality test samples, reinforcing the challenge of domain adaptation in pollen analysis.

By contrast, RN50 models (DINO and COCO-pretrained) underperformed across all datasets, exhibiting markedly lower accuracy and F1-scores. These findings highlight the limitations of convolutional architectures in pollen classification and further emphasize the robust feature extraction capabilities of ViT backbones, which play a pivotal role in automated pollen analysis.

### Performance of object detection models

To assess full object detection performance-including both localization and classification-we trained Faster R-CNN models on our automatically generated Hungarian and Swedish datasets and evaluated them across both internal and external test settings. French slides were reserved for external evaluation only and were not used as a training source (see Supplementary Fig. S1). Each model used a Vision Transformer (ViT) backbone-ViT-S, ViT-B, or ViT-L-with a COCO-pretrained ResNet50 (CO-RN50) serving as a convolutional baseline. We also tested a curated variant (CO-RN50-C) trained on expert-cleaned datasets. We limited curation experiments to the CO-RN50 baseline-our most computationally efficient detector (Supplementary Table S4)-to isolate the effect of curation while keeping training budgets tractable.

This evaluation builds on the earlier backbone classification analysis, now introducing localization and stricter generalization tests. As with classification, internal test sets (from the same slides as training data) yielded inflated scores due to high similarity. Therefore, we focus on external test results to better reflect real-world robustness across domain shifts (see Supplementary Fig. S2). Performance metrics are summarized in Table 3, with internal test results in Supplementary, Appendix F (Table S5). External benchmarking includes Hungarian, Swedish, and-where label overlap permitted-French dataset, summarized in Supplementary, Appendix A, Fig. S1.

**Fig. 2:**
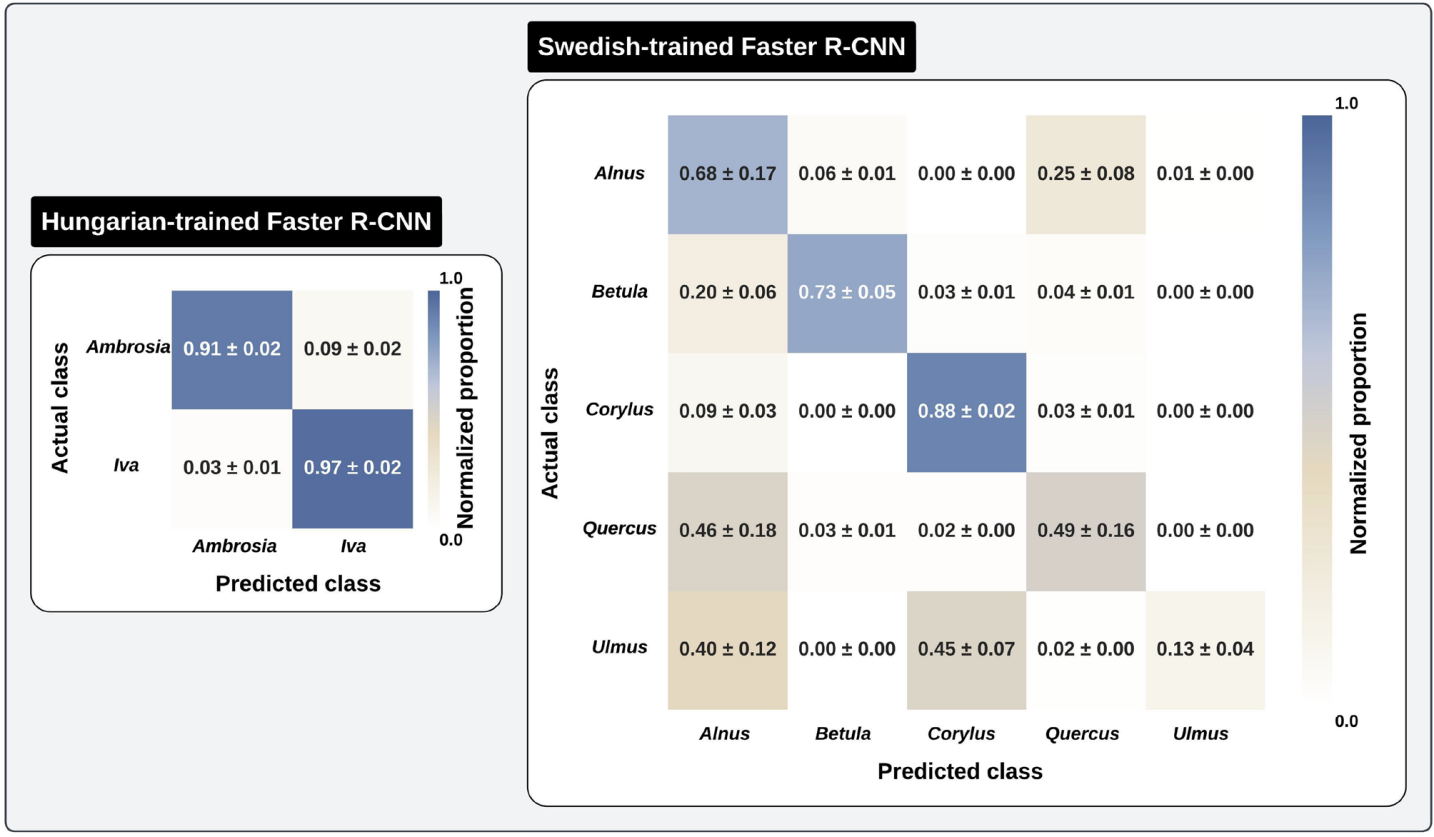
Row-normalized confusion matrices for the best-performing Faster R-CNN models on external test sets. Models were trained on either the Hungarian or Swedish dataset and evaluated on the corresponding external test set, comprising slides from the other region and-where label overlap permitted-France. Each cell represents the mean normalized classification proportion ± standard error across evaluation folds. The Hungarian dataset includes two pollen taxa (*Ambrosia* and *Iva*), while the Swedish dataset includes five taxa (*Alnus*, *Betula*, *Corylus*, *Quercus*, and *Ulmus*).

**Table 3:** Performance metrics for Faster R-CNN object-detection models with various backbones evaluated on external test datasets; models were trained on Hungarian (H) or Swedish (S) sources. Hungarian-trained models were assessed on external slides from Hungary, Sweden, and France, while Swedish-trained models were evaluated on external Swedish and French slides. Results report Average Precision (AP) and Average Recall (AR) at multiple IoU thresholds, averaged across five cross-validation folds with standard error. CO-RN50-C models were trained on a manually curated dataset, whereas all other models were trained on the dataset of highly automated training dataset generation pipeline. Across both datasets, the performance of models trained on automated and curated datasets was highly comparable, with differences within the margin of error. LVD-ViT-L achieved the highest detection accuracy among Hungarian-trained models, whereas CO-RN50 remained competitive in Swedish-trained models, underscoring the impact of dataset quality and domain shifts on model generalization.

Set	Backbone	AP(0.50:0.95)	AP(0.50)	AP(0.75)	AR(0.50:0.95)	AR(0.50)	AR(0.75)
H	LVD-ViT-S	**0.576** ± **0.011**	**0.796** ± **0.005**	**0.741** ± **0.017**	**0.649** ± **0.012**	**0.854** ± **0.005**	**0.807** ± **0.013**
	LVD-ViT-B	0.535 ± 0.009	0.784 ± 0.009	0.721 ± 0.013	0.601 ± 0.007	0.826 ± 0.007	0.777 ± 0.010
	LVD-ViT-L	0.527 ± 0.008	0.787 ± 0.016	0.692 ± 0.008	0.584 ± 0.006	0.816 ± 0.012	0.751 ± 0.007
	UNI-ViT-L	0.162 ± 0.009	0.243 ± 0.013	0.209 ± 0.009	0.343 ± 0.012	0.493 ± 0.013	0.438 ± 0.014
	CO-RN50	0.485 ± 0.014	0.642 ± 0.018	0.624 ± 0.017	0.580 ± 0.009	0.737 ± 0.013	0.722 ± 0.012
	CO-RN50-C	0.479 ± 0.016	0.630 ± 0.022	0.614 ± 0.020	0.578 ± 0.021	0.732 ± 0.027	0.717 ± 0.026
S	LVD-ViT-S	0.112 ± 0.004	0.210 ± 0.008	0.103 ± 0.005	0.216 ± 0.004	0.346 ± 0.005	0.241 ± 0.006
	LVD-ViT-B	0.148 ± 0.002	**0.268** ± **0.003**	0.149 ± 0.002	0.248 ± 0.006	0.393 ± 0.007	0.281 ± 0.008
	LVD-ViT-L	0.152 ± 0.006	0.255 ± 0.011	0.163 ± 0.007	**0.272** ± **0.008**	**0.410** ± **0.015**	0.314 ± 0.009
	UNI-ViT-L	0.086 ± 0.003	0.135 ± 0.008	0.108 ± 0.003	0.144 ± 0.006	0.212 ± 0.013	0.176 ± 0.007
	CO-RN50	**0.159** ± **0.016**	0.236 ± 0.023	**0.216** ± **0.022**	0.245 ± 0.015	0.343 ± 0.017	**0.321** ± **0.017**
	CO-RN50-C	0.164 ± 0.013	0.243 ± 0.020	0.223 ± 0.018	0.258 ± 0.024	0.359 ± 0.033	0.334 ± 0.029

#### Internal test performance

As expected, internal evaluations yielded higher performance across all models compared to external tests, benefiting from domain consistency (Supplementary Table S5). Among Hungarian-trained models, the highest internal average precision (AP@(0.50:0.95)) was 0.697 ± 0.017, achieved by the CO-RN50 model. LVD-ViT-L followed closely with 0.666 ± 0.013. Average recall (AR@(0.50:0.95)) showed a similar pattern, with CO-RN50 reaching 0.732 ± 0.019 and LVD-ViT-L at 0.712 ± 0.012.

Swedish-trained models performed less consistently, with the top AP@(0.50:0.95) of 0.473 ± 0.005 also achieved by CO-RN50. While ViT-based models showed strong feature extraction in earlier classification tasks, they struggled with localization in this more morphologically diverse dataset. Confusion matrices (Supplementary Fig. S5) reveal minimal misclassification in Hungarian models, but higher confusion among *Alnus*, *Betula*, and *Corylus* in Swedish models, highlighting interspecies similarity as a key challenge.

While internal tests help verify that models are learning correctly, they tend to overestimate real-world performance. External evaluations, by contrast, better capture robustness under domain shifts (see Supplementary Fig. S2).

#### External test performance

External evaluations revealed a clear drop in performance compared to internal tests, highlighting the impact of domain shifts (see Supplementary Figures S1 and S2). Among Hungarian-trained models, Faster R-CNN with the LVD-ViT-S backbone achieved the highest AP@(0.50:0.95) at 0.576 ± 0.011, surpassing LVD-ViT-B (0.535 ± 0.009) and LVD-ViT-L (0.527 ± 0.008). All transformer-based models outperformed Faster R-CNN with the CO-RN50 backbone (0.485 ± 0.014), reinforcing the advantage of ViTs for feature extraction in pollen detection. LVD-ViT-S also exhibited the highest recall (AR@(0.50:0.95) = 0.649 ± 0.012), demonstrating its strong detection capabilities.

Swedish-trained models faced greater challenges under domain shift. CO-RN50 reached the highest AP@(0.50:0.95) at 0.159 ± 0.016, followed closely by LVD-ViT-L (0.152 ± 0.006). Despite its strong feature extraction capabilities, LVD-ViT-L demonstrated higher sensitivity to domain shifts, while LVD-ViT-S exhibited the weakest generalization, achieving an AP of only 0.112 ± 0.004. The confusion matrices (Fig. 2) illustrate increased misclassification rates among Swedish taxa, particularly between *Alnus* and *Betula*, as well as *Quercus* and *Ulmus*, suggesting that morphological similarities and inconsistencies in sample preparation impact model performance.

The UNI-ViT-L backbone, pretrained on digital pathology datasets^46^, consistently underperformed across all settings, indicating a pretraining-domain mismatch: features tuned to H&E tissue morphology (e.g., nuclei, glandular/stromal textures, vasculature) transfer poorly to bright-field pollen exine and aperture patterns. These findings underscore that encoder choice is domain-dependent and that greater diversity in training data is needed to improve ViT robustness for multi-class pollen detection; selecting an off-the-shelf encoder from an unrelated domain is insufficient.

Finally, the manually curated baseline (CO-RN50-C) showed performance nearly identical to its automatically generated counterpart. Models trained on curated annotations achieved AP values of 0.479 ± 0.016 (Hungarian test set) and 0.164 ± 0.013 (Swedish test set), closely matching the original (0.485 ± 0.014 and 0.159 ± 0.016, respectively), suggesting that lightweight expert supervision can be a viable substitute for exhaustive manual annotation.

**Table 4:** Performance of Faster R-CNN object detection models with different backbones on real-world airborne pollen samples. Models were trained on the Hungarian dataset and tested on volumetric pollen trap samples to assess real-world applicability. Results report AP and AR metrics at multiple IoU thresholds, averaged across five cross-validation folds. Faster R-CNN with the CO-RN50 backbone achieved the highest AP, demonstrating robustness under operational conditions. Among transformer-based models, LVD-ViT-B exhibited strong performance, while UNI-ViT-L, pretrained on digital pathology data, failed to generalize effectively to airborne pollen detection. CO-RN50-C was trained on a manually curated dataset, whereas all other models were trained using a highly automated training dataset generation pipeline. As in the external validation experiments, models trained on automated and curated datasets achieved comparable performance, with differences within the margin of error.

Set	Model	AP(0.50:0.95)	AP(0.50)	AP(0.75)	AR(0.50:0.95)	AR(0.50)	AR(0.75)
H	LVD-ViT-S	0.326 ± 0.047	0.413 ± 0.062	0.408 ± 0.060	0.355 ± 0.051	0.429 ± 0.063	0.426 ± 0.061
	LVD-ViT-B	0.400 ± 0.037	0.506 ± 0.045	0.494 ± 0.046	0.431 ± 0.039	0.518 ± 0.044	0.511 ± 0.045
	LVD-ViT-L	0.192 ± 0.032	0.269 ± 0.048	0.245 ± 0.040	0.206 ± 0.037	0.271 ± 0.049	0.256 ± 0.045
	UNI-ViT-L	0.012 ± 0.003	0.017 ± 0.004	0.012 ± 0.003	0.013 ± 0.003	0.017 ± 0.003	0.014 ± 0.004
	CO-RN50	**0.559** ± **0.075**	**0.664** ± **0.087**	**0.664** ± **0.087**	**0.599** ± **0.078**	**0.687** ± **0.087**	**0.684** ± **0.087**
	CO-RN50-C	0.541 ± 0.074	0.646 ± 0.087	0.642 ± 0.087	0.584 ± 0.079	0.669 ± 0.089	0.665 ± 0.089

### Real-world validation on air samples

To evaluate model performance in a realistic deployment setting, we tested all detectors on airborne pollen samples collected from Hungary using Hirst-type volumetric traps. This test focused specifically on the detection of *Ambrosia* pollen, one of the most allergenic and ecologically important taxa in Central Europe. Unlike controlled reference slides, these real-world samples exhibited substantial visual variability, including uneven staining, debris, background noise, and dense particle accumulation-conditions that mirror actual operational challenges in national monitoring networks.

Faster R-CNN with the COCO-pretrained ResNet50 (CO-RN50) backbone demonstrated the highest robustness in this setting, achieving an AP@(0.50:0.95) of 0.559 ± 0.075 and an AR of 0.599 ± 0.078 (Table 4). Its curated counterpart, CO-RN50-C-trained on the expert-reviewed dataset-achieved nearly identical results (AP = 0.541 ± 0.074, AR = 0.584 ± 0.079), suggesting that lightweight expert supervision can produce reliable training data for practical applications.

Among transformer-based backbones, LVD-ViT-B was the best-performing ViT model (AP = 0.400 ± 0.037), balancing localization accuracy and classification stability. LVD-ViT-L, despite strong reference-slide performance, exhibited reduced generalization here (AP = 0.192 ± 0.032), indicating sensitivity to environmental noise. The pathology-pretrained UNI-ViT-L performed poorly (AP = 0.012 ± 0.003), reinforcing the need for domain-specific pretraining in ecological image analysis.

## Discussion

We developed a scalable and minimally supervised pipeline for pollen detection that addresses core challenges in training dataset generation, annotation efficiency, and cross-regional generalization for AI-assisted airborne pollen monitoring. By combining open-vocabulary object detection (OWL-ViT)^43^ for one-shot annotation initialization with a streamlined refinement process, our method enables high-throughput dataset assembly with minimal manual effort-critical for advancing pollen analysis at scale.

Benchmarking against expert-curated datasets-created by selectively removing incorrect annotations after automatic generation-confirmed that the automated pipeline maintained low annotation noise (0-3%), validating its reliability. While expert annotation using platforms like Label Studio typically yields 200-600 annotations per hour, our pipeline can produce 10,000-50,000 annotations per hour, representing a major advance toward scalable pollen monitoring. Importantly, this efficiency did not come at the cost of increased human effort: only minimal visual inspection and small classification confidence threshold adjustments were needed to produce high-quality data, and the resulting models performed comparably to those trained on expert-reviewed sets.

Pretrained transformer-based models showed strong feature extraction performance without requiring extensive retraining. LVD-ViT-L achieved up to 87.2% classification accuracy on external datasets, underscoring the potential of general-purpose vision backbones for scalable pollen analysis. However, detection performance varied: while LVD-ViT-S achieved the highest AP@(0.50:0.95) of 0.576 on Hungarian-trained models-outperforming the COCO-pretrained ResNet50 (CO-RN50)-generalization under domain shifts remained a challenge. In Swedish-trained models, CO-RN50 retained the highest accuracy, highlighting the continued importance of dataset adaptation techniques for robust, cross-regional performance.

Real-world validation on airborne pollen samples (*Ambrosia*-only) demonstrated feasibility under field conditions. Detection models trained on both automatically generated and expert-curated datasets achieved comparable accuracy, indicating potential for routine monitoring; however, broader multi-taxon, mixed-sample evaluations will be necessary before considering operational deployment.

While we evaluated multiple backbones and patch sizes, our aim was not to prescribe a single optimal configuration, but to demonstrate the pipeline’s flexibility across different hardware setups and deployment needs. Users can choose model complexity (e.g., ViT-S vs. ViT-L) based on computational resources, throughput requirements, or application-specific constraints. Likewise, patch size can be adapted to slide resolution and memory capacity using standard tools such as OpenSlide. This modular architecture supports broad applicability while allowing for targeted optimization in real-world settings.

Previous studies have relied heavily on manual annotation, which, while accurate, is time-consuming, expensive, and unsuitable for large-scale pollen monitoring^8,39,47^. Semi-automated approaches-typically based on image thresholding or classical OpenCV techniques-improve efficiency but still require extensive expert oversight, limiting scalability^29,40^. In contrast, our pipeline uses a modern open-vocabulary object detector (OWL-ViT)^43^ to eliminate manual bounding-box labeling for training set generation, reducing the number of tunable parameters while preserving annotation quality. This aligns with calls for larger, more diverse datasets to improve model robustness in applied settings^37^. Expert labels were used only to create evaluation ground truth and a lightweight curated baseline by deleting obvious false positives; no manual boxes were added to the training set.

Deep learning in pollen analysis generally follows two paths: (1) classification-based models that analyze single, cropped pollen grains but do not localize them^3,48,49^, and (2) detection models that combine localization and classification in one or two stages^27,28,36,38^. Our approach integrates both, using Faster R-CNN for end-to-end detection, while demonstrating that pretrained ViT backbones-when used with pre-localized input-can support robust classification and generalize well across domains even without fine-tuning.

While some prior studies have used full z-stack imaging to preserve pollen morphology (e.g., Gallardo et al.^28^), this approach is computationally expensive. We instead applied an extended depth-of-field (EDF) method to merge focal planes into a single composite image. This not only reduced storage needs and processing time but also improved clarity, allowing reliable detection with a single optimized layer.

Recent YOLO-based detectors report strong within-domain performance^27,28,50^: on multilabel microscope images of three tree taxa, Kubera et al.^51^ achieved mean average precision (mAP@0.5:0.95) of 0.868-0.924 with YOLOv5, outperforming Faster R-CNN and RetinaNet on the same data; with multifocus (z-stack) acquisition, Gallardo et al.^28^ reported 97.6% grain-location success, 96.3% correct identification, and macro-F1 = 0.956 across 11 taxa. Our study complements these results by automating training-set generation and emphasizing external cross-region evaluation (e.g., Hungary to Sweden/France) and a real-world airborne *Ambrosia* test, thereby reporting non-inflated external scores under domain shift rather than curated laboratory conditions.

Finally, we extended traditional object detection frameworks-such as YOLO^22^ and Faster R-CNN^23,25^-by integrating foundational ViT backbones pretrained on large, diverse datasets^31,52^. This preserved generalization strength while minimizing training costs, and our results from Hungarian-trained models show that transformer-based architectures can be both scalable and robust for pollen monitoring tasks.

While our study advances automated pollen detection, several limitations remain. First, data availability constrained geographic and morphological diversity: our Hungarian and Swedish datasets provided a solid starting point but lacked broader biogeographical coverage. Expanding digitized slide archives-across species, regions, and imaging protocols-will be essential for improving generalization and robustness in real-world applications.

Our airborne-sample validation targeted *Ambrosia* only; operational deployment will require simultaneous detection and quantification across co-occurring taxa in mixed samples, which should be addressed in follow-up studies building on this framework.

Domain shifts introduced by variations in staining, slide quality, and imaging conditions also impacted performance on external tests. While basic normalization techniques (e.g., histogram matching, Macenko staining correction) helped reduce these effects, more advanced domain adaptation approaches may be required for reliable cross-region deployment. At this stage, we prioritized pipeline transparency and reproducibility over implementing more complex unsupervised methods.

Architectural constraints also posed trade-offs. Our ViT-based Faster R-CNN models used plain, non-hierarchical transformer backbones without Feature Pyramid Networks (FPNs), potentially misaligning with the region proposal mechanism and contributing to reduced localization performance in complex settings. Future work could explore hierarchical or multi-scale transformer variants (e.g., Swin^33^) to improve feature compatibility.

Annotation refinement remains an area for improvement. The RGB-based filtering step, while effective at removing outliers, may be less reliable on debris-heavy slides with low pollen-to-noise ratios. Similarly, YOLOS-Tiny offered fast, lightweight refinement but lacked the robustness of larger models-particularly in distinguishing pollen from diverse contaminants. Our use of non-overlapping tiles can miss border objects; future work could adopt overlapping tiles with simple prediction-fusion to recover edge detections. Incorporating feature-guided clustering or more adaptive refinement strategies could further enhance annotation quality.

The performance of OWL-ViT-based one-shot initialization may vary depending on the quality and representativeness of the input exemplars. Although our qualitative inspections showed consistent results, future studies should systematically evaluate exemplar influence on training set precision and downstream model accuracy.

Finally, from an operational standpoint, slide preparation and high-resolution digitization impose fixed time and storage costs. End-to-end full-slide inference is tractable but hardware-dependent and generally requires workstation-class resources (Supplementary Table S4, Supplementary Appendix G).

Future progress in automated pollen detection should target improvements in model architecture, dataset diversity, and deployment-readiness to increase scalability and robustness.

On the architecture side, moving beyond Faster R-CNN to fully transformer-based models-such as Deformable DETR^53^ and Conditional DETR^54^-could eliminate the need for convolutional region-proposal networks and better exploit ViT-style feature extraction^34,44^. Deformable DETR, in particular, offers efficient sparse attention over multi-scale features, which is well suited to detecting small, variably shaped pollen grains. Conditional DETR improves training convergence and detection precision, making it attractive for high-density object settings. Alternatively, a production-oriented hybrid approach could couple a foundational encoder (e.g., DINOv2 ViT) used for pollen-grain localization with a dedicated classifier on candidate regions, fine-tuned on modest, domain-specific labels to capture pollen-specific exine/aperture patterns. This detector-plus-classifier design can be extended with a lightweight vision-language head to support promptable, expert-style queries-resonating with open-vocabulary detection (e.g., OWL-ViT)-while keeping the core pipeline unchanged.

Expanding dataset diversity is critical for generalization. Generating over one million high-quality annotations is feasible through further digitization of pure-species slides and inclusion of laboratory-prepared mixtures that mimic airborne sample complexity. This would expand taxonomic coverage and improve detection performance under varied ecological and environmental conditions.

Foundational models pretrained using self-supervised learning and then fine-tuned on domain-specific data could improve transferability across staining protocols and imaging systems. Nevertheless, caution is warranted: even microscopy-pretrained encoders may not generalize when their pretraining domain diverges from bright-field pollen morphology; for example, the pathology-pretrained UNI-ViT-L underperformed on airborne samples, underscoring the need for domain-specific fine-tuning on pollen imagery. Test-time augmentations and synthetic data generation-such as blending pollen instances into real microscopy backgrounds-may further boost robustness to seasonal and technical variability.

Addressing these future directions will further refine AI-driven pollen detection, advancing toward scalable, automated monitoring for climate, public health, and biodiversity applications, while manual expert identification remains the reference standard for analytical precision.

Near-real-time processing with Hirst-type samplers could enable same-day automated airborne pollen monitoring, reducing analysis time to under three hours post-sample collection. As detailed in Supplementary, Appendix E (Supplementary Table S4), inference times on large scan regions remain tractable even for transformer-based detectors, making deployment feasible at full-slide scale. This would enhance allergen forecasting and environmental reporting, supporting real-time decision-making in public health and ecological research.

By shifting from manual labeling to scalable, AI-assisted dataset generation, our framework dramatically reduces annotation burdens while maintaining opportunities for expert oversight. This transition allows laboratories to modernize workflows, increasing trust in automated systems without compromising analytical precision.

Beyond pollen analysis, the modular structure of our pipeline makes it adaptable to other microscopy-based domains. Potential applications include cancer histopathology, where accurate cell detection informs diagnosis, and microplastic identification for environmental health assessment. These examples underscore the broader relevance of AI-enabled object detection as a scalable, high-precision tool across biological imaging, environmental surveillance, and medical diagnostics.

## Conclusion

We presented a highly automated, end-to-end pipeline for pollen detection that emphasizes scalable training set generation using modern, flexible models such as OWL-ViT for one-shot annotation. Combined with lightweight systematic refinement, this approach produces large-scale, high-quality datasets.

The automated training process was validated across multiple levels-including expert-reviewed annotations, classification of cropped regions, and full object detection-demonstrating both internal consistency and cross-domain generalization. While ViT-based Faster R-CNN models performed well under controlled conditions, domain shifts highlighted challenges in cross-regional robustness. Still, successful deployment on real airborne samples, with accurate *Ambrosia* detection, demonstrated practical feasibility for real-world monitoring.

This work advances AI-assisted pollen analysis toward large-scale, real-world implementation. Future efforts should focus on expanding dataset diversity, refining annotation strategies, and adopting more robust transformer-based architectures to support reliable deployment across varied environmental contexts.

## Methods

**Fig. 3:**
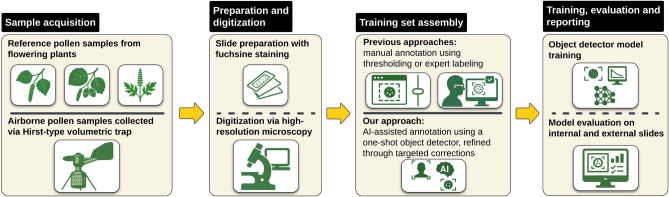
Overview of the automated pipeline for pollen detection. The workflow begins with sample acquisition from two sources: reference pollen collected from flowering plants and airborne samples captured using Hirst-type volumetric traps. Prepared adhesive strips are stained with basic fuchsine and mounted on glass slides, followed by high-resolution digitization. Training datasets are generated using an AI-assisted pipeline that applies a one-shot object detector for initial annotation, refined through targeted corrections-substantially reducing the need for manual labeling compared to traditional thresholding or expert annotation. Trained object detection models are then evaluated on internal and external slides, including real-world airborne samples, to assess performance under diverse conditions.

We developed a minimally supervised pipeline for automated pollen detection that requires no manual bounding boxes during training and only limited expert input for seed exemplars. The workflow is summarized in Fig. 3. The diagram outlines the core stages of our workflow-from sample acquisition and slide preparation to AI-assisted annotation and model evaluation-designed with reproducibility and scalability in mind. The following sections describe each component in detail. We follow the recommended aerobiology terminology for automatic and real-time monitoring^55^.

### Datasets and preprocessing

#### Sources and collection

We compiled datasets from multiple regions, combining pure-species slides with airborne pollen samples. This approach enabled precise species identification while capturing natural environmental variation-crucial for robust model evaluation and scalable ecological monitoring.

The Hungarian dataset focused on two morphologically similar species: *Ambrosia artemisiifolia* (ragweed) and *Iva xanthiifolia* (marsh elder), selected for their close taxonomic relationship and relevance to allergy surveillance. The Swedish dataset included common temperate tree taxa: *Alnus glutinosa* and *Alnus incana* (alder), *Betula pendula* (silver birch), *Corylus avellana* (hazel), *Quercus robur* (English oak), and *Ulmus glabra* (Wych elm). To increase geographic and taxonomic diversity, we incorporated Mediterranean species (*Casuarina*, *Chenopodiaceae*, *Olea*, *Palmaceae*, and *Rumex*) from an open-access source^29^. The Hungarian and Swedish datasets served as the primary training sources for object detection models, while all three datasets were used to evaluate the automated training set labeling process.

To test cross-regional generalization, we used a dataset from France containing *Ambrosia artemisiifolia*, *Betula pendula*, *Quercus robur*, and *Ulmus glabra* as an external validation set. These samples were exclusively used as external reference, were not included in training. In addition, real-world airborne samples were collected in Hungary (2024) using Hirst-type volumetric traps to evaluate performance under natural conditions. For more information, refer to Supplementary Table S2.

In this study, sample quality denotes slide-level and imaging factors that reduce signal-to-noise and boundary clarity - for example, uneven or weak staining; artefacts (debris/background fibres, stain precipitates, air bubbles, scratches, tape/glue edges, and slide cracks); partially broken or collapsed grains; local focus variation from mounting thickness and z-stack fusion; illumination/white-balance differences; and dense overlaps/occlusions-factors that are more variable across the Swedish and Mediterranean scans owing to differences in preparation and imaging protocols.

Throughout this study, we refer to test data originating from the same slide as the training data as *internal*, and test data from other slides, geographic regions, or acquisition protocols as *external*. Specifically, for each slide used in training, a spatially distinct region was withheld and used for internal testing. In contrast, all test slides not used during training-including those from different regions (e.g., France)-are considered *external*. This distinction allows us to evaluate both within-region model performance and cross-region generalization. A detailed summary of dataset composition, imaging resolutions, and their roles in training and evaluation is provided in Supplementary Table S1.

#### Preprocessing techniques

To ensure consistency across datasets while accommodating differences in imaging conditions, we standardized key preprocessing steps. Whole-slide images (WSIs) were processed via focus stacking, patch extraction, and format standardization to optimize image quality and ensure compatibility with model training workflows.

All WSIs were converted to TIFF format, and patching was performed using Python scripts built on OpenSlide and TiffSlide. Patch sizes were selected based on ablation studies and architectural compatibility, particularly aligning with the input dimensions of backbone models used for feature extraction. Sizes such as 518 and 896 pixels were chosen to match the native input resolution of Vision Transformers (ViTs) and Faster R-CNN models while keeping the GPU memory usage manageable. A detailed summary of dataset composition, imaging resolutions, and annotation statistics is provided in Supplementary Table S1, with additional preprocessing steps described in Supplementary, Appendix B.

A core technical contribution of this study is a custom focal-plane stacking algorithm that merges z-stack bright-field microscopy images into a single extended-focus image. Unlike traditional Laplacian-based focus fusion, which may introduce artifacts or fail to preserve morphological detail in dense regions, our method uses a Dual-Tree Complex Wavelet Transform (DTCWT)-based approach^56^. This technique selectively integrates in-focus regions from each slice, preserving sharp structural boundaries while minimizing background noise.

Although DTCWT-based fusion has proven effective in medical and microscopy imaging contexts^57^, its application to large-scale, bright-field pollen datasets has been limited. We adapted and optimized this method for our purposes, with a focus on maximizing image clarity and computational efficiency. The resulting extended-focus images were essential for enabling accurate object detection and classification in our AI models, forming the basis for automated training set generation and evaluation.

### Automated training set assembly

#### Annotation pipeline

**Fig. 4:**
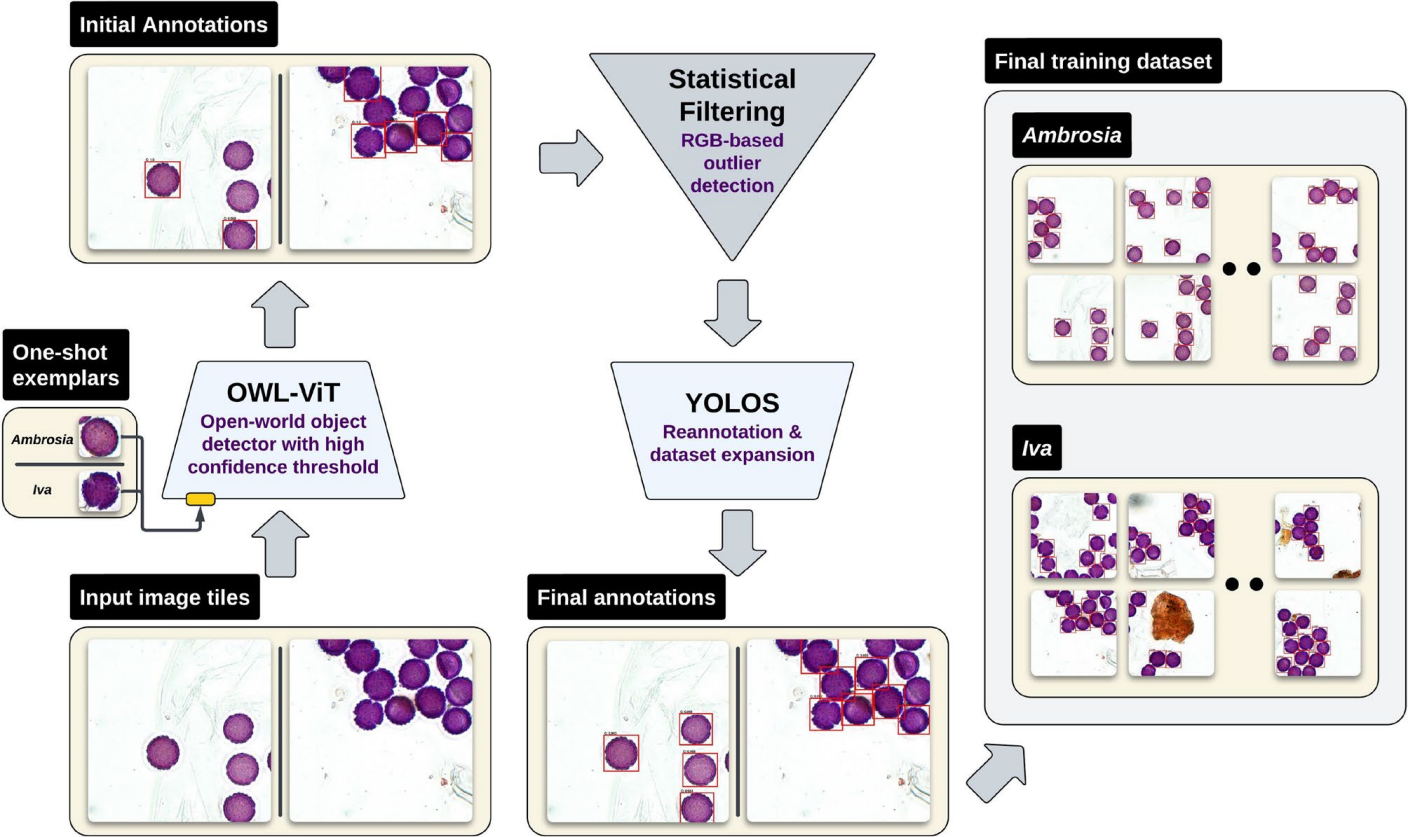
The figure illustrates the full annotation pipeline on two Hungarian reference slides-*Ambrosia* (left lane) and *Iva* (right lane). A single cropped exemplar grain supplies the species label, after which an open-vocabulary detector (OWL-ViT) scans the entire slide and proposes candidate boxes. These boxes are cleaned with a simple RGB-distance filter that discards debris and other artefacts and are then refined by a lightweight YOLOS-Tiny model briefly fine-tuned on the filtered set, recovering missed grains. Because each detection inherits the exemplar’s species label, the pipeline routes all refined boxes directly into species-specific folders, producing thousands of high-quality, fully labelled annotations with no manual box drawing and making large slide archives immediately usable for machine-learning pollen studies. The same annotation pipeline was applied to the Mediterranean and Swedish reference slides, whereas French slides were used only for external evaluation and were not auto-annotated.

To efficiently generate large-scale, high-quality training datasets, we developed a multi-stage annotation pipeline (illustrated on a Hungarian reference pair in Fig. 4). The process begins with OWL-ViT^43^, an open-vocabulary object detector capable of performing one-shot detection using a single exemplar image. For each species, the model is initialized by providing a cropped image of a representative pollen grain-this guides OWL-ViT in identifying visually similar instances across the slide without the need for predefined class training. The same pipeline was applied unchanged to Mediterranean and Swedish slides; French slides served exclusively as external reference data and were not auto-annotated (see Supplementary Fig.  S1).

Applied to non-overlapping tiles of slides, OWL-ViT predicts candidate pollen locations using a high classification confidence threshold, effectively filtering out background debris and minimizing false positives. Since each digitized scan corresponds to a pure-species slide, all detections can inherit the species label of the exemplar used for that slide. This makes the entire OWL-ViT output a weakly supervised but fully labelled dataset for that taxon. The classification confidence threshold-the main hyperparameter-was empirically tuned on a per-slide basis. We used visual inspection to strike a balance between precision and recall: stringent thresholds were applied by default, and selectively relaxed only if detections were sparse or clearly underrepresentative.

Although this approach was exploratory rather than exhaustive, it proved practical for scaling annotation across diverse datasets without the need for manual bounding-box labeling. The process can be initiated by a palynologist or technician within minutes by selecting an exemplar image. The full slide is then processed automatically in minutes to hours, depending on model speed and hardware. We used the base version of OWL-ViT (with a 32-patch Vision Transformer backbone), selected for its efficiency. Under optimal conditions, including a high-performance GPU and streamlined data loading, a full scan could be processed in approximately 30 minutes.

#### Refinement mechanisms

To enhance annotation quality, we implemented a two-step refinement strategy. First, we applied RGB-based statistical filtering to remove visually inconsistent detections. For each candidate box, pixel values were compared to the per-slide class mean RGB vector, and boxes with a Euclidean RGB distance exceeding one standard deviation (1 $\sigma$) were discarded. This threshold was selected based on exploratory plotting and manual inspection, with the aim of prioritizing dataset cleanliness. An example distribution and filtered outlier samples are provided in Supplementary Fig. S4.

Second, YOLOS-Tiny^44^, a lightweight transformer-based detector, was fine-tuned on the RGB-filtered OWL-ViT predictions from each pure-species slide. Each YOLOS model was trained independently for 25 epochs (batch size 32, learning rate 0.001), starting from pretrained weights developed on general object detection benchmarks. Since each training set represented a single taxon with minimal background noise, extensive hyperparameter tuning was not required. The goal of this step was not broad detection, but refinement-removing residual false positives and tightening boxes to produce clean, high-quality annotations for downstream model training.

#### Training set characteristics

Our automated pipeline-combining OWL-ViT initialization with YOLOS-based refinement-produced training datasets with broad coverage and low noise. The iterative design allowed the process to adapt to slide-specific differences while reducing annotation errors, resulting in clean and reliable data for model training.

To evaluate the effect of tile size on annotation stability and output, we conducted an ablation study (see Supplementary Table S3). We tracked the number of detections retained after each stage-initial detection, RGB filtering, and YOLOS refinement-providing insights into how tile size impacts both annotation density and downstream training efficiency.

#### Annotation cleanliness

To evaluate the quality of our automated annotation process, we applied both stages of the pipeline-initial OWL-ViT predictions and subsequent YOLOS-Tiny refinement-to spatially distinct internal test regions that had been withheld from training. These internal tiles, defined earlier as held-out portions of the same slides used in training, were used consistently throughout the study to benchmark performance under realistic but controlled conditions. We compared the resulting annotations from each pipeline stage to high-confidence manual labels created using Label Studio (Supplementary Table S2). This dual evaluation allowed us to quantify the incremental gains from refinement and assess how our annotation generation may work across larger training regions with similar visual and environmental characteristics.

Because both OWL-ViT and YOLOS-Tiny operated on non-overlapping image tiles, we restricted their evaluation on internal test regions to pollen grains with at least 80% of their annotated area contained within a single tile. This exclusion criterion ensured a fair assessment by avoiding penalization for detections of partially visible grains near tile boundaries-instances the models could not reasonably detect. The corresponding performance metrics are reported in the Results section (Table 1).

#### Curated baseline from scalable expert supervision

To better understand how training data quality influences model performance, we created a simplified expert-reviewed version of our automatically generated datasets. This assessment focused not on evaluating model predictions, but on improving the training inputs themselves. After completing the automated annotation and refinement pipeline, we selected two representative tile-based datasets (518$\times$518 and 896$\times$896 pixels) and had experts quickly review them using thumbnail views. Clearly erroneous annotations were removed, but none were added-mirroring how expert workflows often prioritize precision over completeness when time is limited. The full review process required only a few hours per dataset, underscoring the scalability of lightweight expert supervision compared to traditional manual labeling.

We then trained object detection models separately on the unmodified automated datasets and on their curated counterparts. Comparing the two allowed us to quantify the impact of annotation noise on model learning and characterize the trade-offs between full automation and limited expert oversight. The curated datasets thus served as a practical proxy for expert-annotated baselines, helping evaluate how annotation quality affects training rather than test-time evaluation.

For comparability and computational efficiency, we instantiated the curated-data variant only for the standard, widely used Faster R-CNN with a COCO-pretrained ResNet50 backbone (CO-RN50-C); all other backbones were trained on the automated datasets.

### Detection model design and optimization

#### Backbone selection and evaluation

Feature extractors, or backbones, are fundamental to object detection models, shaping how effectively they encode visual patterns and generalize across datasets. In pollen detection, backbone selection directly influences detection accuracy, making it crucial to assess their adaptability and effectiveness. Since foundational models-pretrained on massive image datasets-offer potential shortcuts for rapid deployment and improved cross-origin generalization, we evaluated their performance without requiring extensive retraining or fine-tuning, ensuring their usability for ecological monitoring applications.

To isolate feature extraction quality from detection-specific mechanisms, we reframed the detection task as a classification problem. This controlled evaluation enabled a direct comparison between transformer-based and convolutional backbones under identical conditions. Specifically, we employed a linear probing framework, where each frozen backbone was paired with a single linear classifier trained on pollen image crops. These crops, sourced from Hungarian and Swedish datasets using our automatically generated annotations, were extracted from 896$\times$896-sized tiles and resized to fit backbone-specific input dimensions.

The evaluation included ViT models-ViT-Small (ViT-S), ViT-Base (ViT-B), and ViT-Large (ViT-L)-pretrained on over 100 million images (LVD), alongside a ViT-Large model pretrained on digital pathology tissue data (UNI)^46^. These were compared against two ResNet50 (RN50) convolutional baselines: one trained with self-supervised DINO framework^45^, and another with COCO-pretraining of Faster R-CNN model. This setup provided a direct assessment of transformers’ ability to capture fine-grained pollen morphology compared to established convolutional architectures.

All training and evaluation datasets contained the same pollen taxa, eliminating potential label mismatch effects. The observed performance differences primarily reflect domain shifts introduced by variations in staining, imaging, and sample acquisition. This setup allowed us to evaluate the capacity of foundational vision models to generalize out-of-the-box, without retraining or adaptation, a crucial step toward cost-effective, scalable pollen analysis pipelines.

**Fig. 5:**
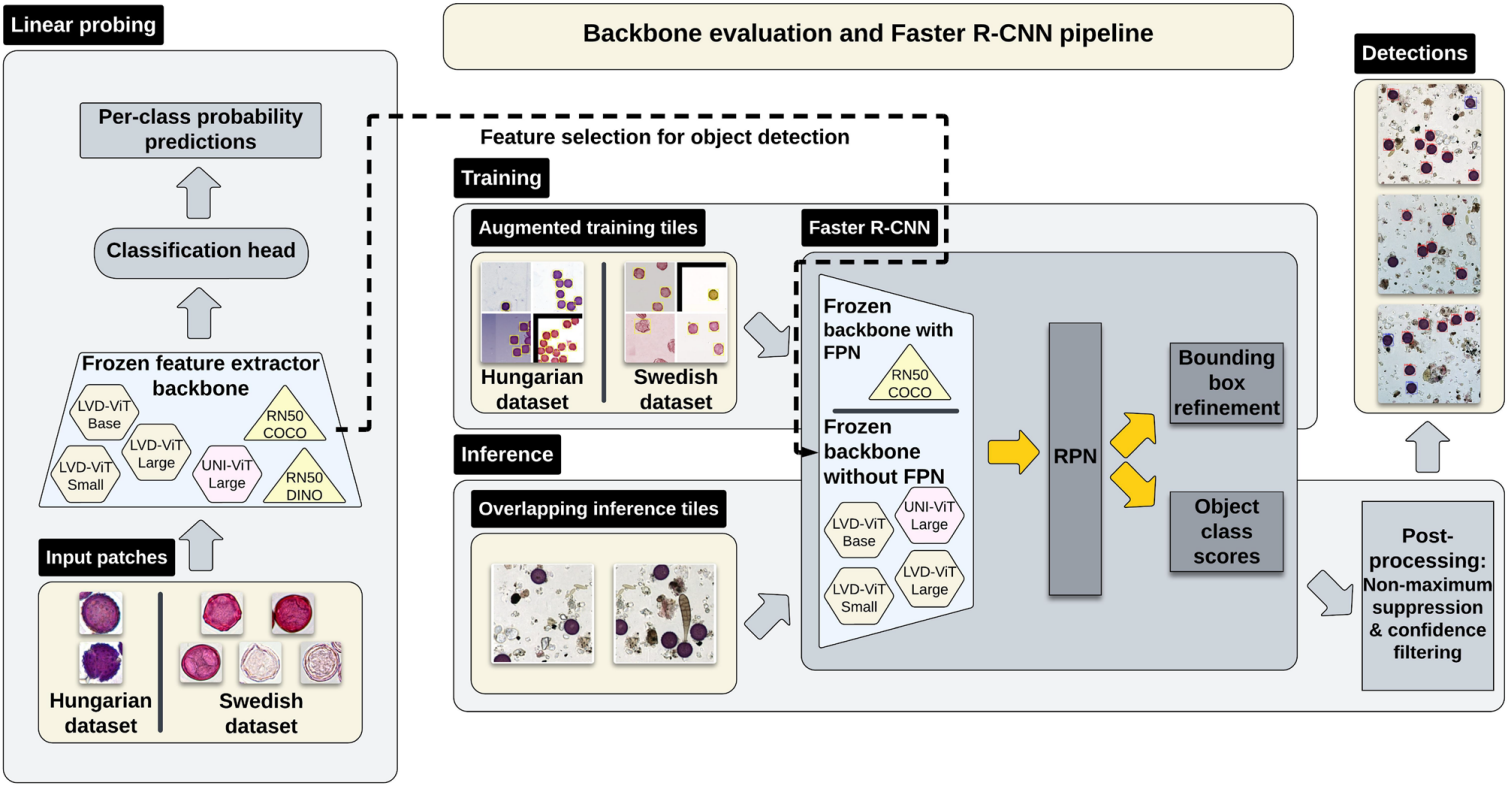
Overview of backbone evaluation and Faster R-CNN integration. Backbone models are first evaluated through linear probing, where per-class probability predictions guide backbone selection for object detection. The best-performing frozen backbones are then integrated into Faster R-CNN, with or without a Feature Pyramid Network (FPN), to enhance multi-scale feature representation. Training is performed on augmented input tiles, while inference leverages overlapping tiles to improve coverage and detection robustness. The Region Proposal Network (RPN) generates bounding box candidates, which are further refined through bounding box regression and object classification. Post-processing steps-including Non-Maximum Suppression (NMS) and classification confidence-based filtering-eliminate redundant detections, ensuring precise pollen localization.

To ensure a fair comparison, we employed cross-validation and balanced subsampling, mitigating dataset biases while maintaining equivalent training iterations across Hungarian and Swedish datasets. Following this, the best-performing backbone was integrated into the Faster R-CNN framework, where its feature extraction capabilities were validated on full detection tasks. The complete pipeline, including backbone selection, Faster R-CNN integration, and inference process, is illustrated in Fig. 5. A detailed breakdown of implementation details, training configuration, and evaluation metrics is provided in Supplementary, Appendix D.

#### Object detection framework

We integrated plain, non-hierarchical ViT backbones into the Faster R-CNN framework^23^, adapting it for automated pollen detection. Faster R-CNN, a widely used two-stage object detection model, was modified to leverage transformer-based feature extraction, addressing the unique challenges posed by pollen grain morphology, scale variation, and fine-grained structures in bright-field microscopy images.

Building on ViTDet’s design principles^52^ and informed by our backbone evaluation results, we replaced traditional convolutional backbones with transformer-based alternatives, specifically ViT-S, ViT-B, and ViT-L. Unlike CNN-based hierarchical designs, this plain-backbone approach processes spatial tokens directly, excluding the CLS token. While conventional Faster R-CNN architectures rely on Feature Pyramid Networks (FPNs) for multi-scale representation learning, we found that ViT backbones inherently capture rich feature hierarchies without explicit pyramid structures in pollen detection. However, for a comprehensive comparison, we included a baseline Faster R-CNN model with a COCO-pretrained ResNet50 (CO-RN50) backbone^25^, serving as a benchmark against transformer-based architectures. This approach preserves computational efficiency while maintaining high detection performance.

To integrate ViT backbones into the Faster R-CNN pipeline, the Region Proposal Network (RPN) and classification heads were adapted to process dense token embeddings. While ViTs lack native hierarchical feature extraction, their feature maps remain structurally compatible with convolutional architectures, allowing seamless integration. A single-scale feature map was used to construct a simplified feature pyramid, preserving fine-grained pollen structures without requiring additional feature fusion mechanisms.

Processing high-resolution digitized scans presents a computational challenge, necessitating image segmentation. To ensure compatibility with ViT’s input dimensions, scans were first divided into larger patches (e.g., 518$\times$518 or 896$\times$896 pixels) and, in some cases, further subdivided (e.g., 224$\times$224) while maintaining spatial context and computational feasibility.

A detailed visualization of the full training and inference pipeline, including backbone selection, Faster R-CNN integration, and post-processing steps, is presented in Fig. 5. Further implementation details, including dataset partitioning, augmentation strategies, and training configurations, are provided in Supplementary, Appendix E.

### Use of AI tools

Portions of this manuscript were supported by the use of AI-assisted writing tools. Specifically, OpenAI’s ChatGPT (version GPT-4o) was used to revise and refine wording, structure, and clarity throughout the manuscript.

All scientific content, experimental design, data analysis, and interpretation were conducted and verified by the authors. The corresponding author takes full responsibility for any AI-generated text or suggestions included in this work. No code was directly generated or deployed from the AI system without manual review and adaptation by the authors.

## Supplementary Information

Supplementary Information.

## Data Availability

All datasets used in this study, including digitized slides and corresponding hand annotations are available upon request. For additional information or specific requests, please contact the corresponding author.

## Code availability

The source code utilized in this study is available from our GitHub repository at https://github.com/abiricz/pollen-auto-annot-init-paper. This repository includes all scripts and comprehensive documentation required to replicate the experiments and evaluations described in this work.
