# Supplementary material for: Efficient and scalable training set generation for automated pollen monitoring with Hirst-type samplers
Source: Sci Rep. 2025 Dec 17;16:2002. doi: 10.1038/s41598-025-31646-2 (PMC12808117; doi:10.1038/s41598-025-31646-2)
Supplement: Supplementary file 1 — Supplementary Information. [file 41598_2025_31646_MOESM1_ESM.pdf]

Supplementary information for: Efficient and scalable training set generation for automated pollen monitoring with Hirst-type samplers

András Biricz[1,*], Donát Magyar[2], Björn Gedda[3], Antonio Spanu[4], János Fillinger[5], Adrián Pesti[6], István Csabai[1], and Péter Pollner[7,8,*]

[1]Department of Physics of Complex Systems, ELTE Eötvös Loránd University, Budapest, Hungary
[2]National Center for Public Health and Pharmacy, Budapest, Hungary
[3]The Palynological Laboratory at the Swedish Museum of Natural History, Stockholm, Sweden
[4]INRAE, UR 546 BioSP, Site Agroparc, Avignon 84914, France
[5]National Korányi Institute for Pulmonology, Budapest, Hungary
[6]Department of Pathology, Forensic and Insurance Medicine, Semmelweis University, Budapest, Hungary
[7]Data-Driven Health Division, National Laboratory for Health Security, Health Services Management Training Centre, Faculty of Health and Public Administration, Semmelweis University, Budapest, Hungary
[8]Department of Biological Physics, ELTE Eötvös Loránd University, Budapest, Hungary
[*]Correspondence and requests for materials should be addressed to *andras.biricz@ttk.elte.hu* or *pollner@emk.semmelweis.hu*

Supplementary Information

Appendix A: dataset information

Our study incorporated a diverse set of datasets, comprising both pure-species slides and real-world airborne pollen samples, to facilitate a comprehensive evaluation under varying imaging conditions. These datasets were collected from multiple geographic regions and processed using standardized imaging and preparation protocols to ensure consistency and comparability across experiments. Table S1 provides a detailed overview of the datasets, including species composition, imaging methodologies, sources, and their designated purposes.

To further illustrate dataset composition and domain shifts, Figure S1 presents representative pollen samples from each dataset. The figure highlights morphological variations between training (Hungarian, Swedish) and evaluation datasets (Hungarian, French, and Swedish), as well as real-world variability in airborne samples, particularly the Hirst-collected *Ambrosia* specimens. To complement this qualitative overview, Figure S2 shows UMAP projections of feature embeddings extracted from test samples using the LVD-ViT-L model. This visualization reveals how domain structure and taxonomic identity manifest in feature space, illustrating both intra-class consistency and cross-domain shifts across geographic sources.

Appendix B: data handling

Preprocessing pipeline and labeling

To ensure consistency across datasets, all raw digitized scans were converted to TIFF format, preserving data integrity and compatibility with downstream processing tools. Given the multi-focal nature of the datasets, focus stacking was applied to generate a single extended-focus image per slide. The Hungarian and French datasets were digitized at 40× and 20× magnifications, respectively, capturing z-stacks of 10 focal planes spaced 1 µm apart. Swedish samples also utilized multi-focal-plane imaging, with some processed using manufacturer-proprietary stacking software and others enhanced using the custom focus-stacking algorithm developed in this study. Mediterranean samples were acquired with an Olympus VS120 microscope, with focus stacking performed using Olympus-proprietary software[1].

To ensure uniform feature extraction while preserving morphological integrity, all datasets were rescaled to a reference magnification of 40×.

Following focus stacking, slides were segmented into smaller subregions optimized for computational efficiency and detection accuracy. Patching was performed using Python-based scripts with OpenSlide and TiffSlide libraries, with patch sizes ranging from 518×518 to 1554×1554 pixels. These sizes were selected based on ablation studies (see Table S3) to identify the optimal resolution for both automated annotation initialization and object detection.

Dataset	Species	Imaging	Source	Purpose
Hungarian	*Ambrosia artemisiifolia*, *Iva xanthiifolia*	3DHISTECH Pannoramic 1000 system, 40× magnification	National Center for Public Health and Pharmacy, Budapest, Hungary	Training and evaluation
Swedish	*Betula pendula*, *Corylus avellana*, *Quercus robur*, *Ulmus glabra*, *Alnus glutinosa and incana*	Objective Imaging system, multi-focal-plane imaging	Swedish Museum of Natural History, Stockholm, Sweden	Training and evaluation
French	*Ambrosia artemisiifolia*, *Betula pendula*, *Quercus robur*, *Ulmus glabra*	3DHISTECH Pannoramic MIDI system, 20× magnification	Réseau National de Surveillance Aérobiologique, Lyon, France	External validation
Mediterranean	*Casuarina*, *Cheno*, *Olea*, *Palmaceae*, *Rumex*	Olympus VS120 microscope, UPLSAPO40x/0.9 objective lens	Public dataset[1]	Demonstrate automated training set generation
Airborne Pollen	Predominantly *Ambrosia artemisiifolia*, collected in late summer pollen season (2024)	3DHISTECH Pannoramic MIDI system, 20× magnification	Collected at National Center for Public Health and Pharmacy, Budapest, Hungary using Hirst-type volumetric pollen traps	Real-world validation

Table S1. Summary of datasets used in this study, including species composition, imaging modality, data source, and intended purpose. The datasets span multiple geographic regions and imaging protocols, supporting cross-regional validation and generalization assessment. The Hungarian and Swedish datasets were used for training and both internal and external evaluations; the French dataset for external validation; the Mediterranean dataset to test annotation initialization on additional pollen taxa; and the airborne samples for real-world performance assessment under natural conditions.

To mitigate staining inconsistencies across datasets, Macenko stain normalization[2] and histogram-matching techniques were applied, ensuring standardized color distributions and minimizing biases introduced by varying staining protocols. This step was particularly crucial for maintaining robust model generalization when training across Hungarian, Swedish, and French datasets.

For external validation, manual ground truth annotations were generated using Label Studio, an open-source labeling tool. This manual labeling was confined to evaluation data only; no manual bounding boxes were drawn for training set generation. This ensured high-quality validation datasets, complementing the automated annotation pipeline and providing a benchmark for evaluating model performance. However, one Swedish external slide—containing a mix of *Alnus*, *Betula*, and *Corylus* pollen—was especially difficult to annotate, as *Betula* and *Corylus* grains are visually similar. As a result, some labels on this slide may include minor inaccuracies, even with expert review.

A detailed summary of the datasets, including species composition, imaging resolutions, and their roles in training, validation, and real-world testing, is presented in Table S2.

Custom focus stacking algorithm for extended depth of field

To improve image quality for model training and evaluation, we implemented a Dual-Tree Complex Wavelet Transform (DTCWT)-based focus stacking algorithm[3,4]. Traditional microscope scanning generates multi-focal-plane z-stacks, capturing both in-focus and out-of-focus regions across different depths. However, proprietary extended depth-of-field (EDF) methods, such as those in 3DHISTECH scanners, often fail to preserve fine-grained structures, introducing ghosting artifacts, misalignment, and inconsistent focus blending that degrade pollen morphology. Given the importance of structural integrity in pollen identification, our approach selectively extracts in-focus regions from multiple focal planes, generating high-fidelity extended-focus images while minimizing visual artifacts.

Our implementation processes z-stacks by first decomposing each focal plane using a multi-scale DTCWT, which separates high-frequency detail and structural edges from background noise. Next, a local energy-based selection is applied across wavelet subbands to retain only the sharpest regions at each pixel location. This approach ensures seamless fusion of in-focus

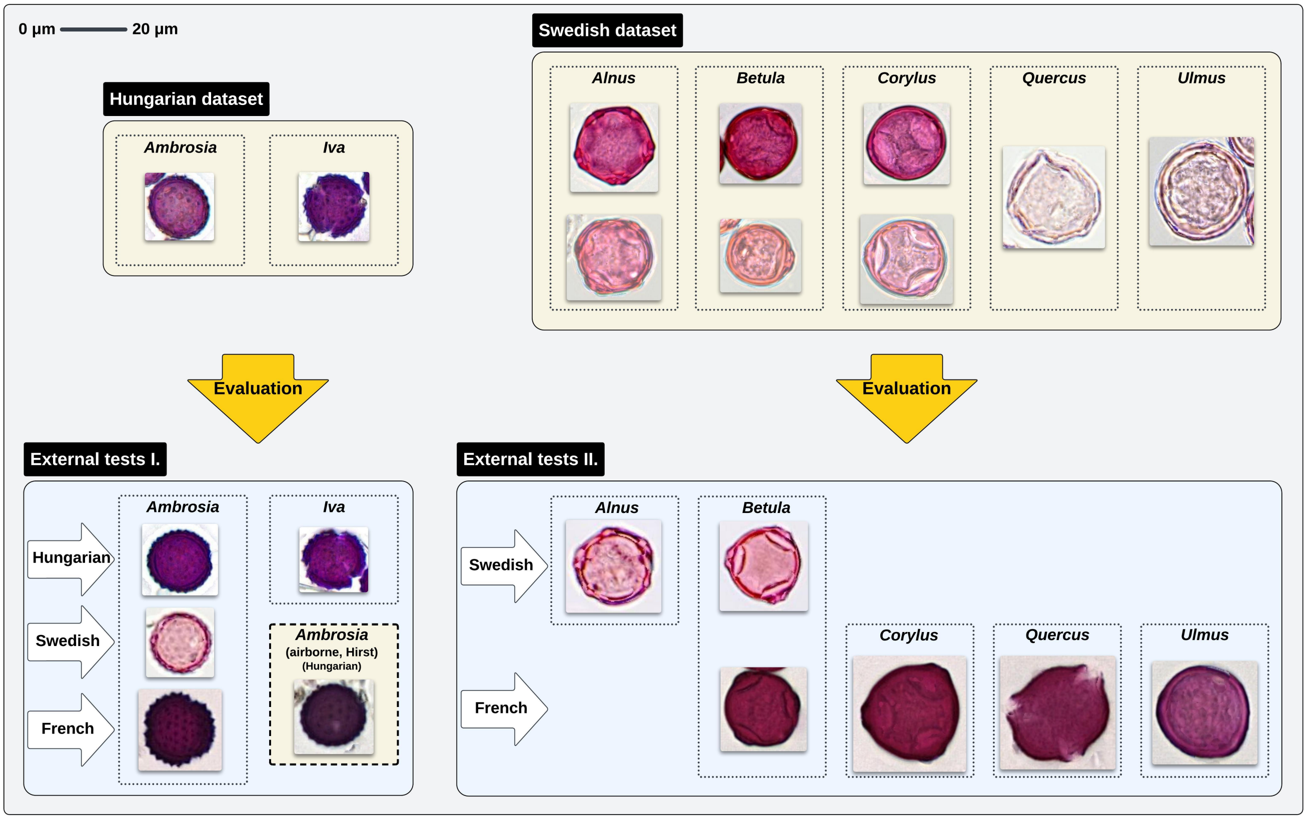

Figure S1. Overview of dataset composition and domain shifts across training and evaluation datasets. Pollen samples from the Hungarian and Swedish datasets were used for model training and internal testing (on held-out regions of the same slides). Evaluation also included external test slides from Hungary, Sweden, and France—unseen during training—and a real-world airborne sample of *Ambrosia* collected using a Hirst-type sampler. Representative pollen images are shown to scale, highlighting inter-regional variation in morphology and staining.

details across depth levels, producing a final stacked image with improved depth representation and minimal haloing or intensity inconsistencies.

Compared to 3DHISTECH's built-in EDF, our custom method significantly reduced blurring and improved structural clarity, particularly in challenging airborne pollen samples, where environmental noise and background complexity typically degrade automated focus stacking (see Figure S3). The DTCWT-based EDF algorithm allowed high-quality dataset generation while maintaining computational efficiency, enabling precise pollen detection and classification across both controlled and real-world airborne samples.

Appendix C: automatic annotation initialization

Automated annotation initialization was performed using OWL-ViT[5], an open-vocabulary object detection framework that generated preliminary bounding boxes from WSIs. Each pollen species was assigned a reference image, enabling OWL-ViT to identify and localize similar structures across the dataset. Annotations were generated at high classification confidence thresholds to reduce false positives.

Table S3 presents the annotation counts across species and patch sizes, illustrating variations in detection performance. These results guided the selection of an optimal patch size to balance computational efficiency with annotation accuracy, ensuring high-quality dataset generation for automated pollen analysis.

Appendix D: backbone evaluation implementation details

To systematically assess the feature extraction capabilities of different backbones, we employed a linear probing approach, a simple classification, ensuring a controlled evaluation of their learned representations. This supplementary details dataset preparation, preprocessing, training setup, and evaluation protocols.

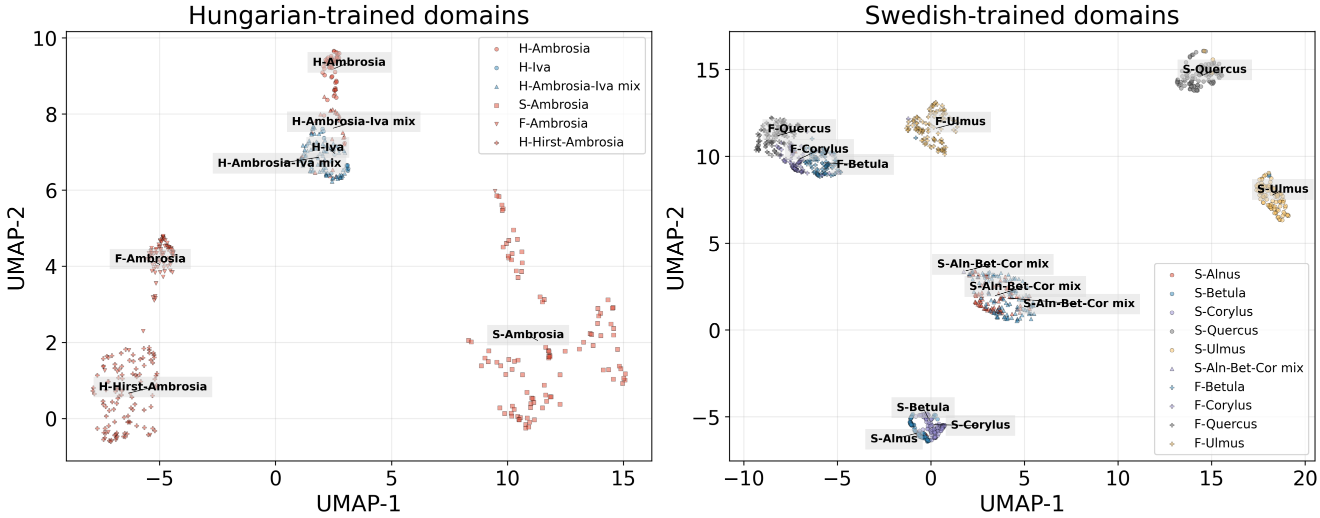

Figure S2. UMAP projections of pollen image features extracted from expert-annotated test regions, plotted from the perspective of Hungarian-trained (left) and Swedish-trained (right) detectors. The plots visualize domain structure, intra-class feature consistency, and cross-domain variability, illustrating how domain shifts emerge across geographic and taxonomic boundaries. Each point represents a pollen crop embedded using the LVD-ViT-L feature extractor. Marker shapes denote sample origin: circles for training slides, upward triangles for additional slides from the same region, and squares, downward triangles, or plus signs for external domains. Colors indicate taxonomic identity, and text labels mark the centroids of major domain clusters.

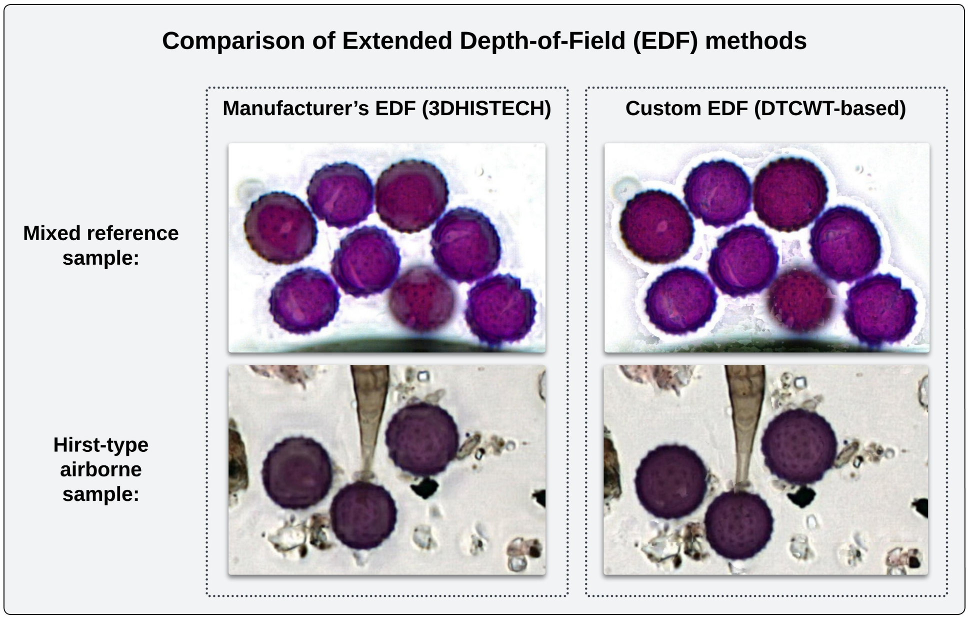

Figure S3. Comparison of extended depth-of-field (EDF) methods applied to pollen microscopy images. The manufacturer’s EDF (3DHISTECH) introduces artifacts and inconsistent focus blending, whereas the proposed DTCWT-based EDF enhances structural clarity and sharpness. Two representative sample types are shown: (Top) mixed reference slide and (Bottom) Hirst-type airborne sample, demonstrating improved feature preservation with the custom EDF method.

Dataset preparation and preprocessing

Pollen image crops were extracted from digitized scans based on the automatically generated annotation set, using 896×896 tiles. Since pollen grain sizes range from approximately 80–100 to 200–250 pixels, each crop was center-cropped and resized to

Dataset	Species	Resolution (pixels)	Magnification	Test	Annotations
Hungarian	*Ambrosia*	110,000 x 120,000	40x	Internal	55
Hungarian	*Iva*	130,000 x 120,000	40x	Internal	17
Swedish	*Alnus (incana)*	392,292 x 40,560	40x	None	None
Swedish	*Alnus (glutinosa)*	30,238 x 30,228	40x	Internal	17
Swedish	*Betula* (#1)	391,914 x 40,520	40x	None	None
Swedish	*Betula* (#2)	371,370 x 40,600	40x	None	None
Swedish	*Betula* (#3)	30,238 x 30,228	40x	Internal	85
Swedish	*Corylus* (#1)	370,732 x 40,560	40x	None	None
Swedish	*Corylus* (#2)	30,238 x 30,228	40x	Internal	185
Swedish	*Quercus*	21,120 x 21,124	20x	Internal	636
Swedish	*Ulmus*	18,176 x 33,184	20x	Internal	476
Mediterranean	*Casuarina*	51,375 x 57,575	40x	Internal	60
Mediterranean	*Chenopodiaceae*	36,325 x 33,700	40x	Internal	797
Mediterranean	*Olea*	56,800 x 25,600	40x	Internal	24
Mediterranean	*Palmaceae*	48,575 x 56,175	40x	Internal	217
Mediterranean	*Rumex*	51,925 x 23,025	40x	Internal	248
Hungarian	*Ambrosia*, *Iva*	7,500 x 7,500	40x	External	127
Swedish	*Ambrosia*	7,500 x 7,500	20x	External	632
Swedish	*Alnus*, *Betula*, *Corylus*	7,500 x 7,500	20x	External	965
French	*Ambrosia*	7,500 x 7,500	20x	External	97
French	*Betula*	7,500 x 7,500	20x	External	411
French	*Corylus*	7,500 x 7,500	20x	External	93
French	*Quercus*	7,500 x 7,500	20x	External	556
French	*Ulmus*	7,500 x 7,500	20x	External	676
Hungarian	Predominantly *Ambrosia*	7,500 x 7,500	20x	Real world test	349

Table S2. Summary of all digitized pollen datasets used in this study for training, internal validation, external evaluation, and real-world testing. The table includes sample origin (by region), species identity, image resolution, magnification level, test set designation (internal, external, or real-world), and the total number of manually verified annotations.

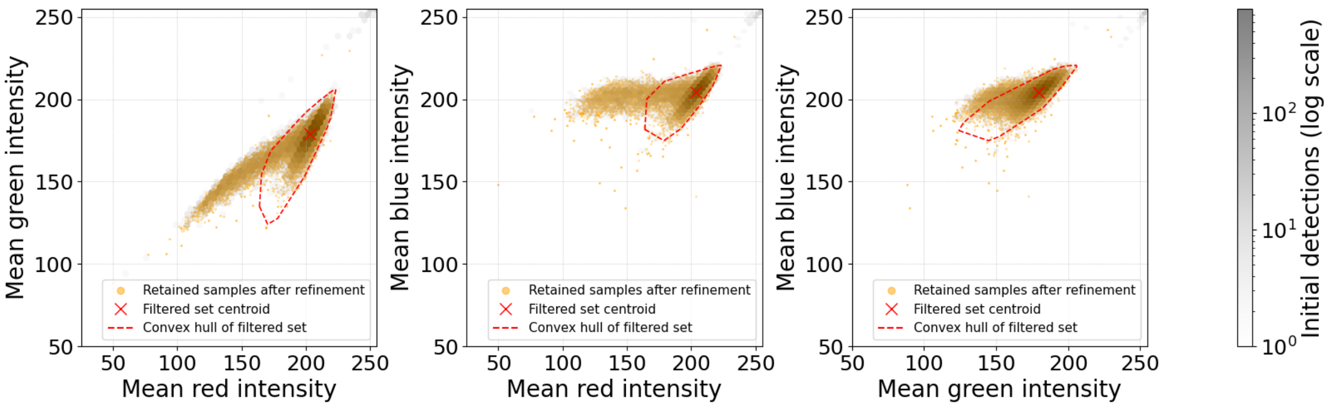

Figure S4. RGB-based statistical filtering and refinement used in the training set assembly for *Ambrosia* pollen. Each subplot shows a two-dimensional projection of the three-dimensional RGB mean feature space. The grayscale hexbin background represents the log-scaled density of initial OWL-ViT detections. The red dashed line outlines the convex hull of the filtered samples, and the red cross marks their centroid. Orange points represent detections retained after refinement using YOLOS-Tiny. A similar procedure was applied to all other species.

224×224 pixels, with padding applied where necessary to maintain aspect ratios. This ensured uniformity across all backbone

Patch Size	Stage	Aln.	Amb.	Bet.	Cor.	Iva	Que.	Ulm.	Cas.	Che.	Olea	Pal.	Rum.
	Initial	5879	15637	13288	17706	14234	1246	1778	1077	4174	980	1512	1025
518	Filtered	4823	11621	10613	12326	10224	1175	1072	795	2444	602	1268	835
	Refined	5787	14931	12336	17357	13655	1256	1642	722	3759	810	2689	1252
	Initial	6794	16702	14758	19746	14608	1331	1958	1115	4473	1069	1392	776
672	Filtered	5383	11817	11113	13254	10550	1253	1224	861	2589	685	1150	650
	Refined	6668	15956	14156	19848	14041	1447	1945	955	3759	989	2975	1088
	Initial	7605	17787	15777	21681	12811	1466	2285	1120	4743	1159	1579	574
896	Filtered	5678	11954	11670	13969	9238	1396	1265	885	2771	722	1290	461
	Refined	7714	17297	15682	21408	14921	1619	2106	701	4054	487	2588	1495
	Initial	7978	18129	16121	22600	10638	1430	2299	1111	4850	1152	1565	528
1036	Filtered	5840	11914	11654	14472	7639	1371	1303	874	2853	724	1221	321
	Refined	7672	17053	16012	22207	13495	1533	2164	1024	4091	1023	2526	47
	Initial	8265	18277	16323	22993	9631	1408	2413	1163	5106	1194	1629	454
1120	Filtered	6024	11939	11404	14857	6884	1337	1263	896	3081	756	1284	277
	Refined	8178	17830	16370	22696	12490	1699	2437	1081	4444	229	2290	0
	Initial	8749	18280	16958	23633	7054	1330	2355	1131	5319	1285	1485	394
1344	Filtered	6259	11539	11750	14822	4968	1096	1324	888	3195	791	1223	209
	Refined	8630	17010	17209	23216	10853	1416	2319	1060	4470	0	2216	0
	Initial	9117	18388	17399	24172	5252	1381	2554	1153	5306	1309	1316	422
1554	Filtered	6489	11481	11913	15315	3633	1288	1358	875	3070	821	1005	372
	Refined	9020	16456	16685	23596	8299	1907	2582	343	4168	0	565	0

Table S3. Comprehensive summary of automated dataset assembly across all species and patch sizes. Counts are presented for Initial annotations (generated by OWL-ViT), Filtered annotations (after statistical filtering), and Refined annotations (post-correction using YOLOS).

architectures.

To mitigate staining inconsistencies and imaging variations, a two-step normalization process was applied. Macenko stain normalization[2] was first used to standardize color distributions across Hungarian, Swedish, and French datasets. At evaluation time, histogram matching aligned the mean and standard deviation of test set images with those of the training set, ensuring consistency in color characteristics.

To ensure balanced training, Hungarian and Swedish datasets were dynamically subsampled to similar scales, maintaining equivalent iteration counts per epoch. This prevented dataset size disparities from biasing model performance while preserving species diversity.

Training protocol and hyperparameters

Each frozen backbone was coupled with a single linear classifier and trained for 50 epochs using a batch size of 1, optimizing classification through frequent gradient updates. The Adam optimizer was employed with a learning rate of $5 \cdot 10^{-5}$, adjusting only the classifier while keeping backbone parameters fixed.

A five-fold cross-validation strategy was implemented, ensuring robustness and preventing overfitting. Stratified sampling preserved class distributions across folds, enabling a fair and reliable comparison between different backbone architectures.

Evaluation metrics

Backbone performance was evaluated using accuracy, precision, recall, and F1-score, providing a comprehensive assessment of classification performance across species. Accuracy measured overall correctness, while precision and recall assessed the model's ability to distinguish species, particularly in handling class imbalances. The F1-score provided a balanced metric, particularly relevant for datasets with skewed distributions. These metrics were computed separately for internal and external validation datasets to assess both in-domain performance and cross-domain generalization.

Appendix E: object detection implementation details

The Faster R-CNN framework with ViT-based backbones was trained using a standardized pipeline to ensure robustness across diverse imaging conditions. This supplementary details the preprocessing steps, augmentation strategies, and training

configurations, including hyperparameter optimization.

Data preprocessing and normalization

To address staining variations and enhance domain consistency, Macenko stain normalization[2] was applied to all training images. At inference, histogram matching was performed to align test set statistics with those of the training distribution. Specifically, for each test WSI, 100 randomly sampled tiles were used to compute mean and standard deviation, ensuring stable normalization across datasets.

The input tile processing pipeline was adapted to accommodate the specific architectural requirements of each backbone. For LVD-pretrained ViT models (ViT-S, ViT-B, ViT-L), 518 × 518 pixel tiles were directly fed into the backbone, aligning with their optimal input dimension. In contrast, the UNI-ViT-L model, which internally operates on 224 × 224 pixel patches, processed 896 × 896 pixel tiles by dividing them into 224 × 224 patches. These patches were batched, sequentially passed through the backbone, and reconstructed into a unified feature map to preserve spatial alignment for downstream integration with the RPN. For convolutional backbones such as CO-RN50, the entire tile was processed directly without subdivision, reflecting the native compatibility of convolutional architectures with larger input dimensions.

Augmentation strategies

A comprehensive augmentation pipeline was implemented to enhance generalization across diverse pollen morphologies and imaging conditions. Geometric augmentations included random resized cropping, flipping, affine transformations, and elastic distortions to simulate variations in pollen orientation and structure. Photometric augmentations, such as color jitter, contrast adjustments, Gaussian blur, sharpening, and ISONoise simulation, preserved structural fidelity while increasing feature diversity. Bounding box integrity was maintained throughout all augmentations to prevent localization errors.

Training configuration

A five-fold cross-validation strategy was adopted to ensure unbiased evaluation, with balanced subsampling applied across Hungarian and Swedish datasets. Each model was trained for 50 epochs using the AdamW optimizer[6], with a tuned learning rate of 1×10^{-4}, which provided optimal stability and convergence. Augmentations were applied dynamically to enhance dataset variability during training.

Anchor box configurations were optimized for pollen morphology, with anchor sizes set to 192 pixels and aspect ratios of $\{0.5, 1.0, 2.0\}$. This choice was informed by a hyperparameter search, as detailed below.

Hyperparameter optimization

To optimize the object detection pipeline, a hyperparameter search was conducted for learning rate and anchor box size, using the ViT-Small Faster R-CNN model due to its computational feasibility. A grid search was performed over learning rates $\{5 \times 10^{-5}, 1 \times 10^{-4}, 2 \times 10^{-4}, 5 \times 10^{-4}\}$ and anchor box sizes $\{128, 192, 256\}$.

Each configuration was trained for 50 epochs using five-fold cross-validation. Learning rate variations had a limited impact on performance within a stable range, but values exceeding 1×10^{-4} led to increased training instability and variance, making 1×10^{-4} the optimal choice.

Anchor size had a more significant effect on detection accuracy, with 192 pixels yielding the highest Average Precision at IoU 0.50:0.95. The 128-pixel anchors underperformed in detecting larger pollen grains, while 256-pixel anchors were less effective for smaller instances, reducing overall robustness. These results guided the final model configuration, optimizing detection performance while maintaining computational efficiency.

Evaluation metrics

To evaluate object detection performance, we used standard metrics from the COCO benchmark: Average Precision (AP) and Average Recall (AR). AP measures the area under the precision–recall curve and reflects the model's accuracy in both localization and classification. AR captures how completely the model detects objects across thresholds.

We report AP and AR at three levels:

- AP@0.50 or AP(0.50) or AP50 – precision at an Intersection over Union (IoU) threshold of 0.50,
- AR@0.50 or AR(0.50) or AR50 – recall at an IoU threshold of 0.50,
- AP@0.75 or AP(0.75) – a stricter IoU threshold of 0.75 for precision,
- AR@0.75 or AR(0.75) – a stricter IoU threshold of 0.75 for recall,
- AP@(0.50:0.95) or AP(0.50:0.95) – the average precision over 10 IoU thresholds from 0.50 to 0.95, in 0.05 increments, providing the most comprehensive score.

- AR@(0.50:0.95) or AR(0.50:0.95) – the average recall over 10 IoU thresholds from 0.50 to 0.95, in 0.05 increments, providing the most comprehensive score.

These notations are used consistently throughout the text and tables to represent performance under increasingly strict localization conditions. These metrics ensure a robust and fine-grained evaluation of model performance across different datasets, including reference slides and real-world airborne samples.

Inference timings for object detection models

To assess the real-world deployment performance of our object detection pipeline, we benchmarked inference times across multiple model and dataset configurations. Evaluation was performed on a 15,000×15,000 pixel region using a 50% sliding window overlap, simulating the processing of a representative high-resolution scan region.
Timings reflect full forward passes of pre-trained models after dataset initialization, excluding training and post-processing. All experiments were conducted using an NVIDIA RTX 4090 GPU with 24 GB VRAM and a batch size of 8. Input patches were streamed from HDD storage via the OpenSlide backend to replicate practical throughput constraints in deployment.
These results scale linearly with slide size. For instance, real Hirst-collected slides can exceed 180,000×60,000 pixels (at 20x magnification). Given this, inference time on such full-resolution slides can be approximated by scaling the reported 15,000×15,000 pixel benchmark by a factor of 50. This translates to approximate end-to-end inference durations ranging from 15 minutes to over 1.5 hours, depending on the model.

Model	Dataset patch size	Time (s)	Relative factor
CO-RN50	896 px	17	1.0×
UNI-ViT-L	896 px	75	4.4×
LVD-ViT-S	518 px	25	1.5×
LVD-ViT-B	518 px	40	2.4×
LVD-ViT-L	518 px	95	5.6×

Table S4. Inference time for a 15,000×15,000 pixel region with 0.5 overlap using various object detection models. The relative factor is normalized to the CO-RN50 baseline. Timings represent end-to-end inference and can be linearly scaled for full slide dimensions. All experiments used an RTX 4090 GPU and OpenSlide-based patch extraction from HDD storage.

Appendix F: extended results

Internal test evaluations provide a controlled assessment of model performance, minimizing the influence of domain shifts. As shown in Table S5, models trained on the Hungarian dataset achieved strong detection performance, with the Faster R-CNN utilizing CO-RN50 backbone obtaining the highest AP@(0.50:0.95) at 0.697 ± 0.017, followed closely by LVD-ViT-Large at 0.666 ± 0.013. Among transformer-based models, LVD-ViT-S exhibited strong localization capabilities, achieving an AP of 0.641 ± 0.007 and an AR of 0.697 ± 0.006, reinforcing the competitive performance of ViTs in controlled settings.
Performance in the Swedish dataset was lower, with the highest AP@(0.50:0.95) at 0.473 ± 0.005 (CO-RN50). LVD-ViT-L followed with 0.415 ± 0.010, but all transformer-based models displayed greater variability, highlighting the increased complexity of multi-class detection in the Swedish dataset. The row-normalized confusion matrices (Figure S5) further illustrate class-specific detection performance, revealing increased misclassification between *Alnus* and *Betula*, as well as *Quercus* and *Ulmus*, suggesting that morphological similarities and dataset diversity affect model discrimination.
These results indicate that while internal evaluations establish a strong performance baseline, they do not fully reflect real-world conditions.

Appendix G: reproducibility

Computational resources

All experiments were conducted on high-performance infrastructure to ensure efficient processing of large-scale datasets.

Scanning and normalization As a reference, scanning time for samples from the French dataset at 20× with 10 focal planes was approximately 30–40 minutes per slide on a 3DHISTECH scanner. Using the vendor software, each slide required an additional ∼30 minutes to convert scans into standardized TIFF files.

Preprocessing Data preparation—including focus stacking and annotation refinement—was executed on an AMD Threadripper CPU (32 cores), leveraging multithreading for whole-slide image processing. Focus stacking times were on a par with scanning and conversion, at ∼40 min per slide using 32 CPU cores.

Set	Model	AP(0.50:0.95)	AP(0.50)	AP(0.75)	AR(0.50:0.95)	AR(0.50)	AR(0.75)
H	LVD-ViT-S	0.641 ± 0.007	0.877 ± 0.016	0.806 ± 0.016	0.697 ± 0.006	0.894 ± 0.017	0.849 ± 0.006
	LVD-ViT-B	0.642 ± 0.005	0.935 ± 0.002	0.866 ± 0.013	0.692 ± 0.004	0.951 ± 0.002	0.893 ± 0.011
	LVD-ViT-L	0.666 ± 0.013	**0.945 ± 0.002**	0.887 ± 0.018	0.712 ± 0.012	**0.952 ± 0.000**	0.912 ± 0.014
	UNI-ViT-L	0.598 ± 0.013	0.903 ± 0.007	0.823 ± 0.024	0.646 ± 0.013	0.909 ± 0.006	0.854 ± 0.017
	CO-RN50	**0.697 ± 0.017**	0.930 ± 0.023	**0.905 ± 0.024**	**0.732 ± 0.019**	0.933 ± 0.023	**0.917 ± 0.024**
S	LVD-ViT-S	0.388 ± 0.011	0.812 ± 0.008	0.255 ± 0.026	0.448 ± 0.011	0.825 ± 0.007	0.415 ± 0.028
	LVD-ViT-B	0.403 ± 0.008	0.841 ± 0.016	0.276 ± 0.014	0.464 ± 0.009	0.850 ± 0.016	0.441 ± 0.013
	LVD-ViT-L	0.415 ± 0.010	**0.881 ± 0.007**	0.255 ± 0.020	0.481 ± 0.009	**0.891 ± 0.006**	0.439 ± 0.025
	UNI-ViT-L	0.367 ± 0.007	0.684 ± 0.017	0.303 ± 0.014	0.401 ± 0.008	0.688 ± 0.017	0.414 ± 0.011
	CO-RN50	**0.473 ± 0.005**	0.860 ± 0.008	**0.439 ± 0.024**	**0.516 ± 0.005**	0.865 ± 0.008	**0.566 ± 0.016**

Table S5. Performance metrics for object detection models on internal test sets for Hungarian and Swedish datasets. Results include AP and AR metrics at varying IoU thresholds, averaged across 5 cross-validation folds. Best-performing models for each metric are highlighted.

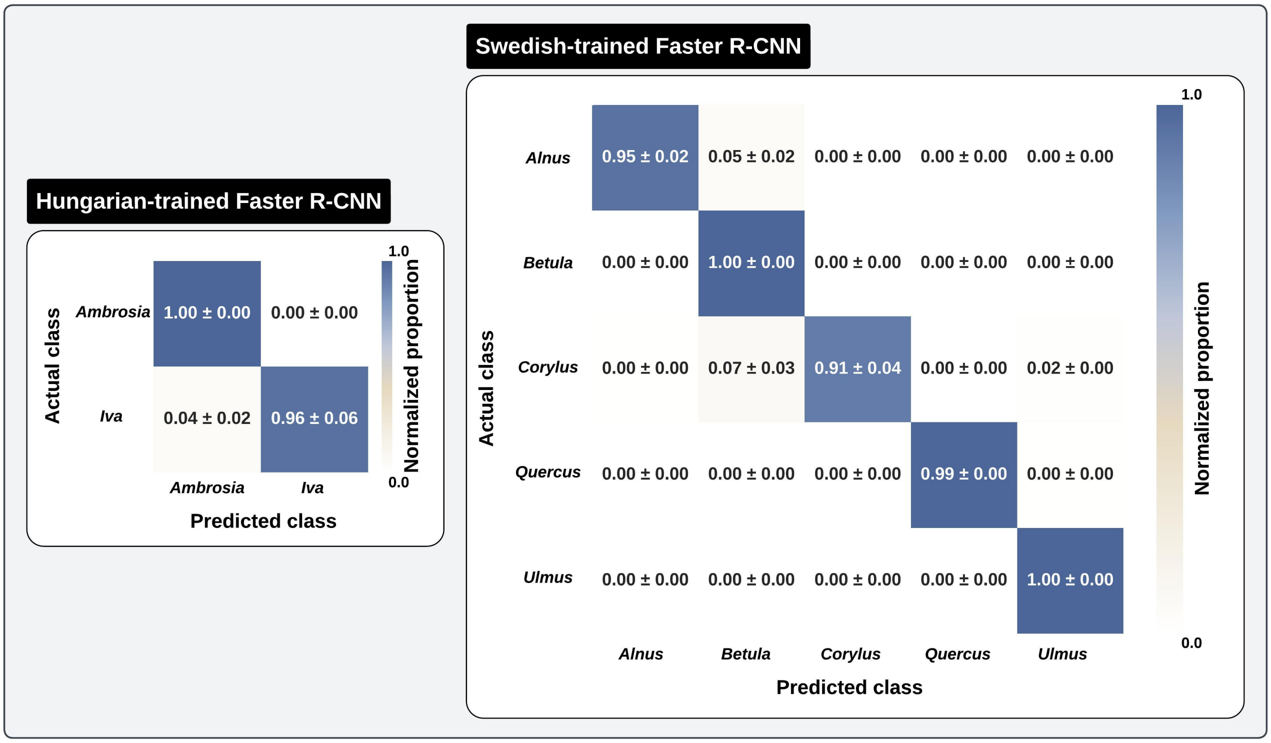

Figure S5. Row-normalized confusion matrices for the best-performing Faster R-CNN models evaluated on internal test sets. Each model was tested on its respective test dataset. Values represent mean normalized classification proportions ± standard error across evaluation folds. The Hungarian-trained model (left) was evaluated on Hungarian data (*Ambrosia*, *Iva*), while the Swedish-trained model (right) was tested on Swedish data (*Alnus*, *Betula*, *Corylus*, *Quercus*, *Ulmus*).

Model training Object-detection and backbone-evaluation models were developed and trained on an NVIDIA GeForce RTX 4090 GPU. Training times ranged from 1 to 20 hours per run, depending on model complexity and input resolution.

Scalability considerations Large-scale experiments and hyperparameter tuning leveraged a dedicated server with eight NVIDIA A100 GPUs at the Wigner GPU Laboratory. This infrastructure facilitated rapid experimentation with high-resolution image inputs, significantly reducing training time for ViT-based Faster R-CNN models.

All models were implemented in PyTorch with CUDA acceleration. Measured per-model inference timings and hardware

details are summarized in Appendix E Table S4. Timings are indicative and depend on slide area, number of focal planes, I/O throughput, and model/backbone choice.

References

1. Chaves, A. J. *et al.* Pollen recognition through an open-source web-based system: automated particle counting for aerobiological analysis. *Earth Sci. Informatics* **17**, 699–710, DOI: 10.1007/s12145-023-01189-z (2024).

2. Macenko, M. *et al.* A method for normalizing histology slides for quantitative analysis. In *2009 IEEE International Symposium on Biomedical Imaging: From Nano to Macro*, 1107–1110, DOI: 10.1109/ISBI.2009.5193250 (2009).

3. Selesnick, I., Baraniuk, R. & Kingsbury, N. The dual-tree complex wavelet transform. *Signal Process. Mag. IEEE* **22**, 123 – 151, DOI: 10.1109/MSP.2005.1550194 (2005).

4. Ravi, J. & Narmadha, R. Optimized dual-tree complex wavelet transform aided multimodal image fusion with adaptive weighted average fusion strategy. *Sci. Reports* **14**, 30246, DOI: 10.1038/s41598-024-81594-6 (2024).

5. Minderer, M. *et al.* Simple open-vocabulary object detection. In *Computer Vision – ECCV 2022: 17th European Conference, Tel Aviv, Israel, October 23–27, 2022, Proceedings, Part X*, 728–755, DOI: 10.1007/978-3-031-20080-9_42 (Springer-Verlag, Berlin, Heidelberg, 2022).

6. Loshchilov, I. & Hutter, F. Decoupled weight decay regularization. In *International Conference on Learning Representations* (2017).
